# Influence of Substrate Concentration on the Culturability of Heterotrophic Soil Microbes Isolated by High-Throughput Dilution-to-Extinction Cultivation

**DOI:** 10.1128/mSphere.00024-20

**Published:** 2020-01-29

**Authors:** Ryan P. Bartelme, Joy M. Custer, Christopher L. Dupont, Josh L. Espinoza, Manolito Torralba, Banafshe Khalili, Paul Carini

**Affiliations:** a Department of Environmental Science, University of Arizona, Tucson, Arizona, USA; b Department of Environment and Sustainability, J. Craig Venter Institute, La Jolla, California, USA; c Department of Ecology and Evolutionary Biology, University of California, Irvine, California, USA; U.S. Department of Energy Joint Genome Institute

**Keywords:** genome streamlining, microbial cultivation, oligotrophy, soil microbial ecology

## Abstract

The vast majority of microbes inhabiting oligotrophic shallow subsurface soil environments have not been isolated or studied under controlled laboratory conditions. In part, the challenges associated with isolating shallow subsurface microbes may persist because microbes in deeper soils are adapted to low nutrient availability or quality. Here, we use high-throughput dilution-to-extinction culturing to isolate shallow subsurface microbes from a conifer forest in Arizona, USA. We hypothesized that the concentration of heterotrophic substrates in microbiological growth medium would affect which microbial taxa were culturable from these soils. To test this, we diluted cells extracted from soil into one of two custom-designed defined growth media that differed by 100-fold in the concentration of amino acids and organic carbon. Across the two media, we isolated a total of 133 pure cultures, all of which were classified as *Actinobacteria* or *Alphaproteobacteria*. The substrate availability dictated which actinobacterial phylotypes were culturable but had no significant effect on the culturability of *Alphaproteobacteria*. We isolated cultures that were representative of the most abundant phylotype in the soil microbial community (*Bradyrhizobium* spp.) and representatives of five of the top 10 most abundant *Actinobacteria* phylotypes, including *Nocardioides* spp., *Mycobacterium* spp., and several other phylogenetically divergent lineages. Flow cytometry of nucleic acid-stained cells showed that cultures isolated on low-substrate medium had significantly lower nucleic acid fluorescence than those isolated on high-substrate medium. These results show that dilution-to-extinction is an effective method to isolate abundant soil microbes and that the concentration of substrates in culture medium influences the culturability of specific microbial lineages.

**IMPORTANCE** Isolating environmental microbes and studying their physiology under controlled conditions are essential aspects of understanding their ecology. Subsurface ecosystems are typically nutrient-poor environments that harbor diverse microbial communities—the majority of which are thus far uncultured. In this study, we use modified high-throughput cultivation methods to isolate subsurface soil microbes. We show that a component of whether a microbe is culturable from subsurface soils is the concentration of growth substrates in the culture medium. Our results offer new insight into technical approaches and growth medium design that can be used to access the uncultured diversity of soil microbes.

## Abstract (precis)

Isolating environmental microbes and studying their physiology under controlled conditions are essential aspects of understanding their ecology. Subsurface ecosystems are typically nutrient-poor environments that harbor diverse microbial communities—the majority of which are thus far uncultured. In this study, we use modified high-throughput cultivation methods to isolate subsurface soil microbes. We show that a component of whether a microbe is culturable from subsurface soils is the concentration of growth substrates in the culture medium. Our results offer new insight into technical approaches and growth medium design that can be used to access the uncultured diversity of soil microbes.

## INTRODUCTION

Soil microbial communities are tremendously diverse and mediate crucial aspects of plant fertility, biogeochemistry, pollutant mitigation, and carbon sequestration (1–4). While the diversity and community composition of surface soils have been relatively well described, we know far less about the microbes inhabiting deeper soils (defined here as >10 cm below the surface), despite their key roles in soil formation and mineralization of key plant nutrients. In contrast to surface soils that are typically rich in plant-derived compounds, subsurface soils are often characterized by smaller amounts of mineralizable nitrogen, phosphorus, and organic carbon—much of which has a long residence time and is relatively recalcitrant to microbial degradation (5–9). The temperature and soil moisture of subsurface soils are also less variable than those of shallower soils that are exposed to seasonal changes in temperature and precipitation (10). These relatively stable and low-nutrient conditions found at depth constrain both the amount of microbial biomass present in the subsurface and the structure of these microbial communities (11–14). Many of the microbial taxa that are abundant in these subsurface environments are underrepresented in microbial culture and genome databases (11). Thus, there are large knowledge gaps in our understanding of the biology of a major fraction of subsurface soil microbes.

Because subsurface soils are low-nutrient habitats, part of the challenge associated with culturing and studying the microbes that live belowground may be that they require low nutrient concentrations in order to be isolated or propagated in the laboratory (15). These microbes—often referred to as “oligotrophs”—are capable of growing under conditions where the supply or quality of nutrition is poor. Although oligotrophs dominate most free-living microbial ecosystems (16), the concept of oligotrophy itself is enigmatic. There is no coherent definition of what constitutes oligotrophic metabolism aside from their ability to grow at “low” nutrient concentrations—a definition that itself is arbitrary (17, 18). Kuznetsov et al. (19) identified three groups of cultivatable oligotrophs: (i) microbes that can be isolated on nutrient-poor medium but cannot be subsequently propagated, (ii) microbes that can be isolated on nutrient-poor medium but can be subsequently propagated on nutrient-rich medium, and (iii) microbes that require special nutrient-poor medium for both isolation and propagation. Although the molecular and genetic mechanisms that distinguish these three categories are poorly understood, several traits of oligotrophs have emerged from the study of microbes that numerically dominate oligotrophic ecosystems. For example, oligotrophs are typically small, slowly growing cells (20–23). The genome sizes of numerous lineages of microbes that dominate oligotrophic marine ecosystems tend to be highly reduced—an indication that microbial oligotrophy may be tied to reduction of genome size (24–26). These “streamlined” genomes often code for fewer copies of the rRNA gene operon and transcriptional regulator genes than microbes with larger genomes, suggesting that oligotrophs lack the ability to sense and rapidly respond to variable environmental conditions (12, 16, 92). Instead, genomic inventories of marine oligotrophs suggest a reliance on broad-specificity, high-affinity transporters that are constitutively expressed (22, 26–28).

While the activities of abundant and ubiquitous microbes that inhabit oligotrophic marine environments have been extensively investigated in recent years (24, 29, 30), far fewer studies have focused on the activities of microbes that dominate oligotrophic soil environments. Several soil studies used low-throughput techniques to show that reduced-nutrient solid media facilitated the isolation of important soil microbes that were previously uncultured (31–34). While several agar-based high-throughput approaches have been used to isolate diverse microbes (35, 36), these approaches may not be appropriate to isolate microbes that thrive at micromolar amounts of growth substrate and do not form detectable colonies on solid media. Here, we adapt existing high-throughput dilution-to-extinction protocols, originally developed for isolating abundant aquatic oligotrophic bacteria, to facilitate the isolation of soil microbes. We hypothesized that the concentration of heterotrophic growth substrates in a growth medium would constrain which taxa were able to be isolated on a custom-designed defined artificial medium. We tested this by extracting cells from oligotrophic subsurface soils using Nycodenz buoyant density centrifugation (37) and inoculating high-throughput dilution-to-extinction experiments in two defined media that contained a 100-fold difference in the amounts of heterotrophic growth substrates. We isolated several bacteria that were representative of abundant phylotypes found in the original soil microbial community and two lineages representative of uncultured groups of microbes. In these experiments, the substrate concentration significantly influenced which actinobacterial genera were culturable in the laboratory but had no effect on which alphaproteobacterial lineages were culturable. Moreover, we show that cells isolated on low-nutrient medium had significantly lower SYBR green I nucleic acid fluorescence, suggesting that microbes isolated on low-nutrient medium may contain reduced nucleic acid content relative to those isolated on higher-nutrient medium.

## RESULTS

**FIG 1:**
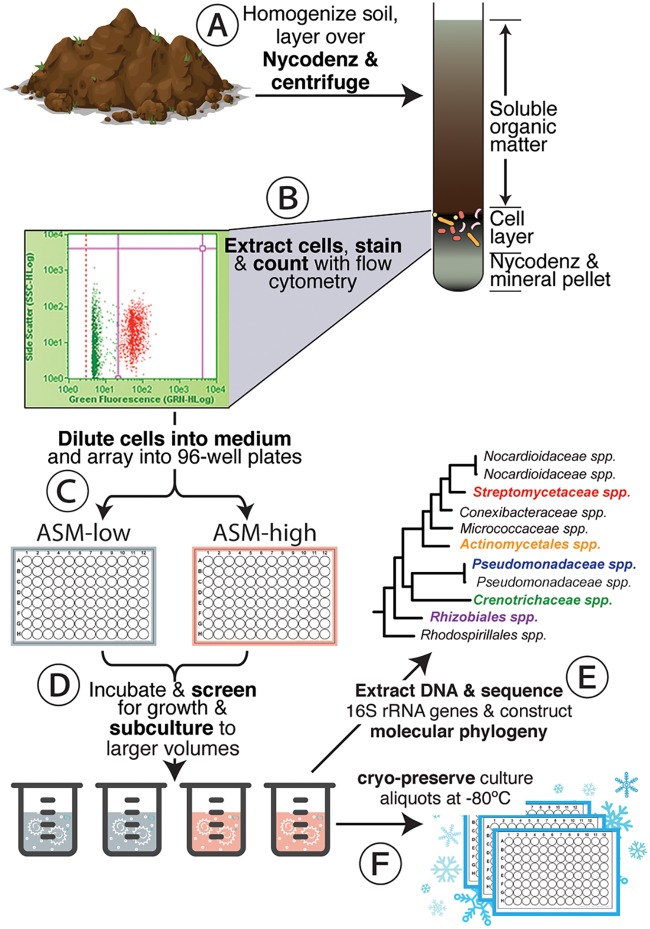
Dilution-to-extinction workflow. Soils were collected and brought to the lab, where they were homogenized in cell extraction buffer, layered over a Nycodenz solution, and centrifuged (A). The cell layer was extracted from the Nycodenz solution and counted with flow cytometry (B). Counted cells were diluted into growth medium in 96-well microtiter plates to an average density of 5 cells well^−1^ (C). After incubation, the 96-well microtiter plates were screened for growth with flow cytometry, and wells displaying growth were subcultured into larger volumes (D). After incubating the subcultures, flasks displaying growth were identified by 16S rRNA gene sequencing and molecular phylogeny (E). Aliquots of these identified subcultures were cryopreserved at −80°C (F).

We collected shallow subsurface soil (55 cm) from the Oracle Ridge field site in a midelevation conifer forest that is part of the Santa Catalina Mountains Critical Zone Observatory in Arizona, USA. These soils contained very small amounts of total organic carbon (0.095%) and N-NO_3_ (0.3 ppm), indicating they were highly oligotrophic (see Fig. S1 in the supplemental material). We adapted existing high-throughput dilution-to-extinction approaches designed for aquatic microbes (38, 39) to culture soil microbes from these samples (Fig. 1). The primary modification to existing protocols was to add a buoyant density centrifugation cell separation step to detach inoculum cells from mineral soils prior to diluting cells into growth medium. To do this, we vortexed soil in a cell extraction buffer containing a nonionic surfactant and a dispersing agent. We layered this soil-buffer slurry over 80% Nycodenz and centrifuged it. During centrifugation, the mineral components of soil migrated through the Nycodenz, while cells “floated” on the surface of the Nycodenz. We extracted cells located at the Nycodenz interface, stained them with SYBR green I, and counted them on a flow cytometer. This extraction yielded 1.28 × 10^5^ cells ml^−1^ from 0.5 g wet soil. We diluted the extracted cells to an average of 5 cells well^−1^ in deep-well polytetrafluoroethylene 96-well plates containing a custom-designed and defined growth medium that we named artificial subterranean medium (ASM), with low or high concentrations of heterotrophic growth substrates (ASM-low and ASM-high, respectively) (Fig. 1). The ASM-low and ASM-high media contained identical inorganic mineral and vitamin amendments but a 100-fold difference in the concentration of organic carbon and amino acids (Table S1). We designed these media to facilitate the growth of diverse chemoheterotrophic microbes by including an array of simple carbon compounds, polymeric carbon substrates, and individual amino acids (Table S1). We prepared triplicate 96-well plates for each growth medium formulation. These dilution-to-extinction experiments were screened for growth with flow cytometry after 4 weeks of incubation and again after 11 weeks of incubation (Fig. 1). Wells displaying growth (defined as those wells displaying 1.0 × 10^4^ cells ml^−1^) were subcultured into larger volumes and subsequently cryopreserved and identified by 16S rRNA gene sequencing (Fig. 1).

10.1128/mSphere.00024-20.1 FIG S1 The soils used for cultivation were oligotrophic. (A) Points are the measured soil organic carbon content of 184 soil samples collected from shallow subsurface pits located across the United States (from reference 11). (B) Points are the measured NO_3_-N content of 121 soils (113 surface soils) collected across the United States (from references 90 and 91). The organic carbon percent and NO_3_-N measured in our 55-cm sample from Oracle Ridge are illustrated with a red dashed line in both panels. Our sample falls on the low end of both organic carbon content and NO_3_-N content across this broad selection of soils. Download FIG S1, EPS file, 1.3 MB. Copyright © 2020 Bartelme et al. 2020 Bartelme et al. This content is distributed under the terms of the Creative Commons Attribution 4.0 International license.

10.1128/mSphere.00024-20.5 TABLE S1 Artificial subterranean growth medium (ASM)-low and -high formulations including concentrations of carbon sources, amino acids, vitamins, trace metals, and inorganic nutrients. Download Table S1, XLSX file, 0.01 MB. Copyright © 2020 Bartelme et al. 2020 Bartelme et al. This content is distributed under the terms of the Creative Commons Attribution 4.0 International license.

**FIG 2:**
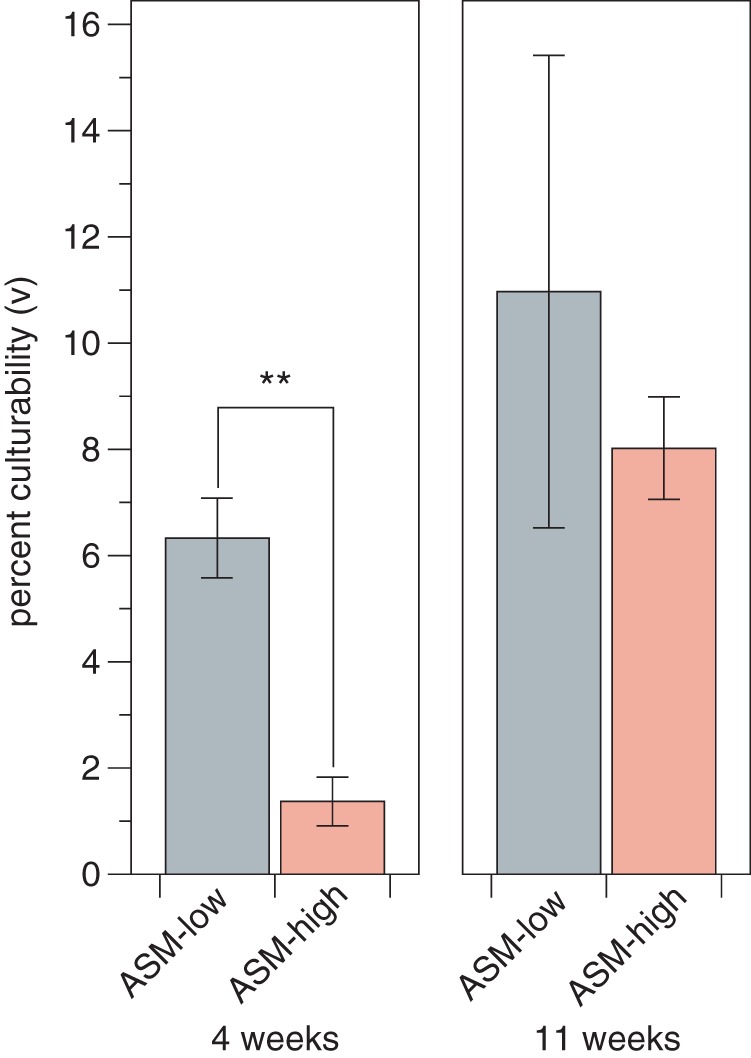
Microbial culturability (*v*) was greater on ASM-low than on ASM-high. Bar heights are the mean percent culturability ± standard deviation in 96-well microtiter plates (*n* = 3) as calculated from the initial cell inoculum and the proportion of wells positive for growth (40). Double asterisks indicate Wilcoxon rank sum test *P* values of ≤0.05.

Across the two medium types, a total of 214 wells (119 for ASM-low and 95 for ASM-high) displayed growth after 11 weeks of incubation. We successfully propagated 182 (85%) of the cultures from microtiter plates to polycarbonate flasks containing fresh medium. Of the cultures that successfully propagated, we confirmed that 73% (133 cultures) were pure cultures by amplifying and sequencing full-length 16S rRNA gene sequences from genomic DNA extractions. The remaining 49 cultures were mixed (forward and reverse 16S rRNA sequence reads did not assemble due to base ambiguities) or, in rare instances, did not amplify under several amplification conditions. We defined microbial culturability using Button’s definition of microbial “viability” as determined in dilution-to-extinction experiments (40). Here, “culturability” is defined as the ratio of cells that grew into detectable cultures to the total number of cells initially diluted into a cultivation chamber (40). The culturability metric described here is informative to evaluate the suitability of a growth medium to isolate microbes and can be applied across medium formulations and experiments so that different experiments can be directly compared. By the end of the experiment, we approached ∼20% culturability across the two medium formulations (Fig. 2). In general, microbial culturability was higher for ASM-low than for ASM-high, but this effect was significant only after 4 weeks of incubation (Fig. 2; Wilcoxon rank sum test, *P ≤ *0.05 at 4 weeks).

**FIG 3:**
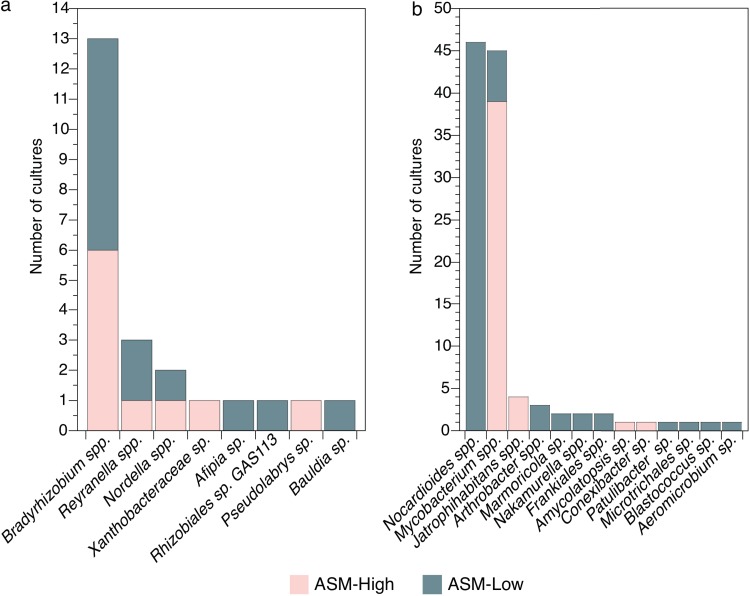
ASM-low and ASM-high cultured distinct *Alphaproteobacteria* (a) and *Actinobacteria* (b). Bar heights are the number of cultures obtained for each taxon and are colored by the medium type on which they were isolated. The genera assigned to bacteria isolated on ASM-low were distinct from those isolated on ASM-high (Kruskal-Wallis rank sum, χ^2^ = 19.05, *P = *1.28 × 10^−5^). These differences were driven by differences in culturability across medium types for actinobacterial genera but not for alphaproteobacterial genera (Dunn test, *P* ≤ 0.000 for *Actinobacteria* and *P* = 0.128 for *Alphaproteobacteria*).

We assigned taxonomy to each 16S rRNA gene sequence using the SILVA database. All pure cultures isolated on ASM-low and ASM-high belonged to one of two bacterial phyla: *Actinobacteria* (110 cultures; 83% of the pure cultures) or *Proteobacteria* (23 cultures, all *Alphaproteobacteria*; 17% of the pure cultures) (Fig. 3). Across all experiments, the genera assigned to bacteria isolated on ASM-low were significantly distinct from those isolated on ASM-high (Kruskal-Wallis rank sum, χ^2^ = 19.05, *P = *1.28 × 10^−5^). However, these differences were largely driven by significant differences in culturability across medium types for the *Actinobacteria* but not for the *Alphaproteobacteria* (Dunn test, *P ≤ *0.000 for *Actinobacteria* and *P = *0.128 for *Alphaproteobacteria*).

Just over half of the *Alphaproteobacteria* (57%) were isolated on ASM-low medium, and the remaining 43% were isolated on ASM-high (Fig. 3 and Fig. S2). Cultures that were classified as *Bradyrhizobium* spp. were the most frequent alphaproteobacterial isolates (13 isolates), seven of which were isolated on ASM-low medium. Cultures classified as *Reyranella* spp. and *Nordella* spp. were also isolated on both ASM-high and ASM-low medium. Of the remaining five *Proteobacteria* cultures, three were isolated on ASM-low (*Afipia* [1 culture], *Rhizobiales* [1 culture], and *Bauldia* [1 culture]), and two were isolated on ASM-high (*Pseudolabrys* [1 culture] and a *Xanthobacteraceae* sp. [1 culture]). The actinobacterial cultures belonged to three classes: *Actinobacteria* (107 cultures), *Thermoleophilia* (2 cultures), and *Acidimicrobiia* (1 culture). Of these *Actinobacteria*, 65 (59%) were isolated on ASM-low, and 45 (41%) were isolated on ASM-high. The cultures were numerically dominated by two genera that were differentially isolated on ASM-low and ASM-high: *Nocardioides* and *Mycobacterium*. *Nocardioides* spp. (46 cultures) were exclusively isolated on ASM-low medium (Fig. 3 and Fig. S3). Other cultures that were isolated on ASM-low included those classified as *Arthrobacter* (3 cultures), *Marmoricola* (2 cultures), *Nakamurella* (2 cultures), *Aeromicrobium* (1 culture), *Blastococcus* (1 culture), and *Patulibacter* (1 culture) (Fig. 3 and Fig. S3). While the majority of cultures classified as *Mycobacterium* sp. were isolated on ASM-high (38 cultures), we isolated seven mycobacterial cultures on ASM-low medium—five of which form a phylogenetically distinct cluster from those isolated on ASM-high (Fig. 3 and Fig. S3). Other actinobacterial cultures isolated on ASM-high included *Jatrophihabitans* (4 cultures), *Conexibacter* (1 culture), and *Amycolatopsis* (1 culture).

10.1128/mSphere.00024-20.2 FIG S2 Alphaproteobacterial IQ-TREE maximum-likelihood full-length 16S rRNA gene sequence phylogeny. The tree was constructed using a GTR+F+I+G4 nucleotide substitution model iterated over 10,000 ultrafast bootstraps. The scale bar indicates the FigTree proportionally transformed branch length. The 16S rRNA gene sequence from E. coli K-12 was used as an outgroup. Clade bootstrap support is indicated by colored nodes as indicated in the legend. This tree includes 16S rRNA gene sequences that amplified from all pure alphaproteobacterial cultures isolated in this study (bolded black leaves denoted with “AZCC”), as well as their best sequence matches to NCBI microbial genomes (dark blue leaves), NCBI 16S rRNA gene sequences from cultured isolates (orange leaves), and NCBI 16S rRNA gene sequences from environmental clones (green leaves). The growth medium concentration on which each AZCC culture was isolated is indicated by colored bars on the right side of the phylogenetic tree (red for ASM-high and blue for ASM-low). Download FIG S2, PDF file, 0.4 MB. Copyright © 2020 Bartelme et al. 2020 Bartelme et al. This content is distributed under the terms of the Creative Commons Attribution 4.0 International license.

10.1128/mSphere.00024-20.3 FIG S3 Actinobacterial IQ-TREE maximum-likelihood full-length 16S rRNA gene sequence phylogeny. The tree was constructed using a SYM+R10 nucleotide substitution model iterated over 10,000 ultrafast bootstraps. The scale bar indicates the FigTree proportionally transformed branch length. The 16S rRNA gene sequence from B. subtilis DSM-10 was used as an outgroup. Clade bootstrap support is indicated by colored nodes as indicated in the legend. This tree includes 16S rRNA gene sequences that amplified from all pure actinobacterial cultures isolated in this study (bolded black leaves denoted with “AZCC”), as well as their best sequence matches to NCBI microbial genomes (dark blue leaves), NCBI 16S rRNA gene sequences from cultured isolates (orange leaves), and NCBI 16S rRNA gene sequences from environmental clones (green leaves). The growth medium concentration on which each AZCC culture was isolated is indicated by colored bars on the right side of the phylogenetic tree (red for ASM-high and blue for ASM-low). Actinobacterial classes are indicated by colored vertical bars to the right of the tree figure: purple for *Thermoleophilia*, teal for *Acidimicrobiia*, and orange-red for *Actinobacteria*. Download FIG S3, PDF file, 0.6 MB. Copyright © 2020 Bartelme et al. 2020 Bartelme et al. This content is distributed under the terms of the Creative Commons Attribution 4.0 International license.

Interestingly, we isolated what are likely the first members of two novel actinobacterial lineages on ASM-low. The first such culture—*Microtrichales* sp. strain AZCC_0197—belongs to the *Microtrichales* order of the *Acidimicrobiia* class. The best 16S rRNA gene sequence match to an existing isolate is 93.4% identity to Aquihabitans daechungensis strain G128. However, strain AZCC_0197 more closely matched numerous 16S rRNA gene sequences from environmental clones of uncultured *Acidimicrobiia*. The second lineage—*Frankiales* sp. strains AZCC_0102 and AZCC_0072—were classified as members of the *Frankiales* order of the *Actinobacteria* class with best matches of <97% nucleotide identity to existing *Frankiales* isolates (41).

**FIG 4:**
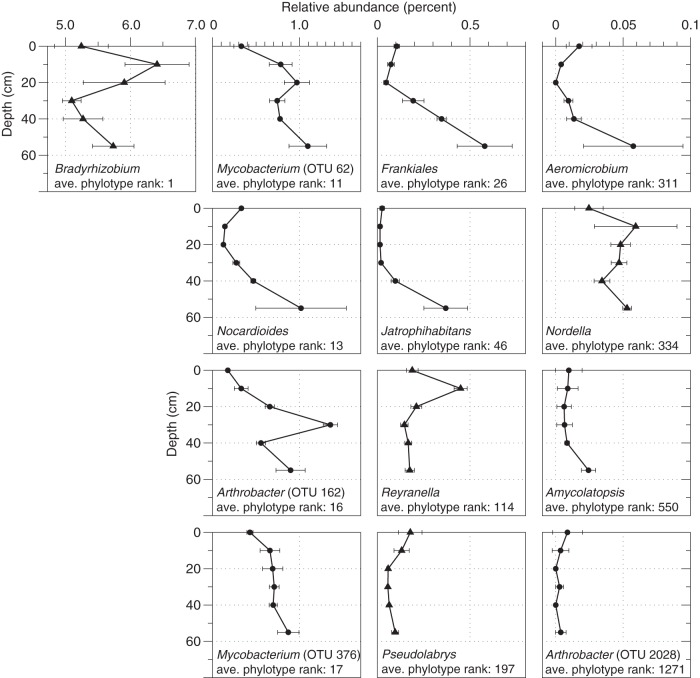
The cultures isolated in this study were representative of several abundant soil lineages that show dynamic depth distributions in Oracle Ridge soils. Points are the mean relative abundances ± standard deviation (*n* = 3) of 16S rRNA gene sequence phylotypes that matched the 16S rRNA gene sequences obtained from cultured isolates at ≥97% identity. Error bars that are not visible are located behind the symbol. Assigned genus names and the average (*n* = 3) relative rank of each phylotype at 55 cm are shown. Cultures classified at the genus level as *Mycobacterium* and *Arthrobacter* cultures matched more than one phylotype in the cultivation-independent surveys. The best-matching OTU number is shown in parentheses. Triangles are *Alphaproteobacteria*. Circles are *Actinobacteria*.

Several of the microbes we isolated were representative of abundant members of the subsurface soil microbial community at the Oracle Ridge site. We matched the 16S rRNA gene sequences from our cultures to the phylotypes derived from the 55 cm Oracle Ridge soil sample. The 16S rRNA gene sequences from our cultures matched 13 phylotypes (at 97% identity [Fig. 4]) that account for 11.0% ± 1.6% (mean ± standard deviation [SD], *n* = 3) of the total amplifiable microbial community. For example, the 16S rRNA gene sequences from our *Bradyrhizobium* isolates match a single *Bradyrhizobium* phylotype that was the most abundant phylotype at 55 cm (relative abundance of 5.7% ± 0.3% [mean ± SD, *n* = 3] [Fig. 4]). Additionally, we isolated representatives of abundant *Actinobacteria* (Fig. 4), including two *Mycobacterium* phylotypes (the 11th and 17th most abundant phylotypes overall), *Nocardioides* (the 13th most abundant phylotype overall), and two *Arthrobacter* phylotypes (16th and 1,271st most abundant phylotypes overall). The other *Actinobacteria* cultured in these experiments represent rarer phylotypes in bulk soils. The 16S rRNA gene sequences from several of our pure cultures did not match any of the phylotypes derived from these soils at ≥97% identity, including *Nakamurella* (2 cultures), *Nocardioides* (5 cultures), *Mycobacterium* (1 culture), *Jatrophihabitans* (1 culture), *Patulibacter* (1 culture), *Conexibacter* (1 culture), *Rhizobiales* sp. (1 culture), *Reyranella* (1 culture), and *Microtrichales* sp. strain AZCC_0197.

**FIG 5:**
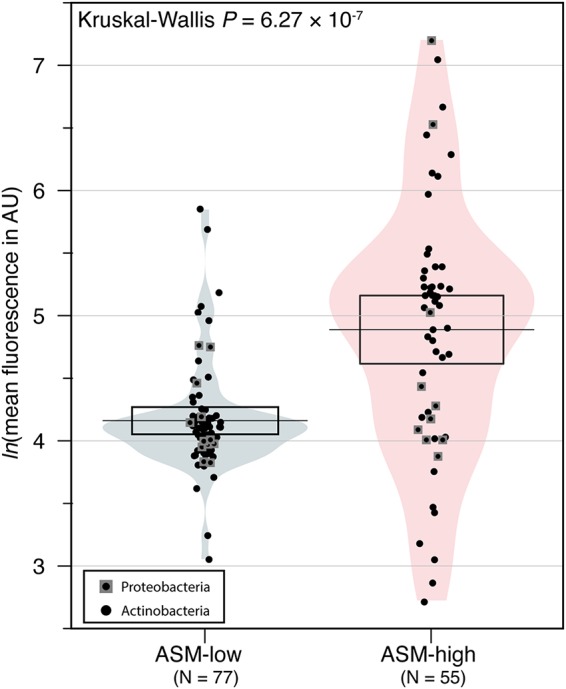
The mean nucleic acid fluorescence of taxa isolated on ASM-low was significantly lower than for those microbes isolated on ASM-high. Points are the mean natural logarithm (ln) of the quantified nucleic acid fluorescence (in arbitrary units [AU]) of fixed and SYBR green I stained stationary-phase cultures. The mean fluorescence value was obtained from manually gated histogram plots of fluorescence within the Guava EasyCyte software. Only those cultures that were defined as pure cultures are plotted. The horizontal line in each plot is the mean fluorescence value, and the box surrounding the mean is a 95% confidence interval. Shading illustrates the relative distribution of fluorescence values within each medium type.

In many environments, relative nucleic acid content can be estimated with flow cytometry analysis of cells stained with nucleic acid-staining dyes (42–44). Given that many microbes inhabiting low-nutrient environments exhibit reduced genome sizes, we sought to determine whether the nucleic acid fluorescence measured for our cultures partitioned by the growth medium on which they were isolated. For each culture, we identified the closest match to an available genome sequence and found that the genome length of these “best hit” matches was significantly correlated with the average fluorescence of SYBR green I stained cells (Spearman’s rho = 0.3, *P = *0.0012), indicating that the nucleic acid fluorescence we quantified by flow cytometry may be indicative of genome size differences. We found that cultures isolated on ASM-low exhibited significantly lower mean nucleic acid fluorescence than those isolated on ASM-high (Fig. 5) (Kruskal-Wallis rank sum χ^2^ = 24.8, *P = *6.27 × 10^−7^). The overall mean fluorescence was not significantly different across the phyla assigned to each isolate (Kruskal-Wallis rank sum χ^2^ = 0.210, *P = *0.647) but was significant across individual genus assignments (Kruskal-Wallis rank sum χ^2^ = 62.4, *P = *2.98 × 10^−6^). Moreover, the mean nucleic acid fluorescence values within a given genus were similar (Fig. S4). For example, *Mycobacterium* isolates had relatively high nucleic acid fluorescence, regardless of which medium they were isolated on (Fig. S4). In contrast, *Nocardioides* (ASM-low) and *Jatrophihabitans* (ASM-high) displayed relatively low nucleic acid fluorescence. Interestingly, we observed a clear nucleic acid fluorescence dichotomy across the *Bradyrhizobium* isolates isolated on ASM-high (Fig. S4).

10.1128/mSphere.00024-20.4 FIG S4 Distribution of nucleic acid fluorescence values by phylum (a) and genus (b). Data are natural logarithm-transformed nucleic acid fluorescence values of fixed and SYBR Green I-stained stationary-phase cultures. Plots show all genera with *n* ≥ 3 cultures in one or both culture medium types. Shaded regions illustrate the distribution of fluorescence values within culture medium groups. Download FIG S4, EPS file, 1.5 MB. Copyright © 2020 Bartelme et al. 2020 Bartelme et al. This content is distributed under the terms of the Creative Commons Attribution 4.0 International license.

## DISCUSSION

We designed a proof-of-concept workflow to determine the feasibility of high-throughput dilution-to-extinction cultivation for the isolation of soil microbes. The method was based on a workflow for isolating microbes from oligotrophic marine environments (38). However, unlike aquatic samples, microbial cells in soils are heterogeneously dispersed within, or attached to, a complex matrix comprised of noncellular organic matter and minerals. The complexity of this soil matrix complicates accurate enumeration of viable cells because mineral and organic matter can interfere with flow cytometry. To circumvent these issues, we separated cells by gently shaking soils in a cell extraction buffer containing a dispersing agent and a nonionic surfactant. Cells were separated from this slurry by buoyant density centrifugation (Fig. 1). This procedure allowed cells to be floated on top of a dense solution of Nycodenz while allowing minerals to migrate through the Nycodenz solution (45).

We estimated the expected culturability and calculated the actual culturability using the statistical framework of dilution culture growth outcomes described by Button et al. (40). The expected number of pure cultures (*û*) was estimated across all experiments using the formula *û* = −*n*(1 − *p*) × ln(1 − *p*), where *p* is the proportion of wells displaying growth (214 growth chambers displaying growth/576 chambers inoculated = 0.37) and *n* is the number of inoculated chambers (576 chambers in total). Based on this equation, we expected *û *= 168 pure cultures across all experiments. The number of pure cultures we obtained (133 cultures) was within 21% of this value. However, this result is conservative because it does not account for the cultures that were initially scored as positive for growth but could not be successfully subcultured. Some of these cultures may be oligotrophic taxa that were initially cultivatable but failed to successfully propagate, as described by Kuznetsov et al. (19). Alternatively, cultures that failed to propagate from microtiter plates to larger volumes might have been false positives, where flow cytometer instrument noise or well-to-well carryover was mistaken for a low-density culture. The mean culturability we observed for a given experiment (1.4% to 11% [Fig. 2]) was comparable to dilution-to-extinction cultivation studies of marine microbes, which report 0.5% to 14.3% culturability (39). Similar to previous observations for soil microbes (33), we observed increased culturability with longer incubation times (Fig. 2). We speculate that the culturability was higher on ASM-low than on ASM-high because the cells were extracted from an extremely oligotrophic soil sample (see Fig. S1 in the supplemental material), and perhaps the results would have been different if the original inoculum originated from more productive soils.

The concentration of heterotrophic growth substrates in the isolation medium significantly influenced our ability to isolate certain actinobacterial lineages (Fig. 3). In particular, *Nocardioides* were exclusively isolated on ASM-low and most *Mycobacterium* isolates were isolated on ASM-high. We designed our media to include a defined but diverse range of carbon substrates that have been successfully used to isolate chemoheterotrophic microbes from soil or oligotrophic taxa from other environments (33, 46). Organic carbon type and availability are crucial for heterotrophic soil microbes because carbon acts as both a source of electrons for respiration and a nutrient for growth. To accommodate this requirement, many common microbial growth medium formulations for heterotrophic microbes supply diverse growth substrates (yeast extract or casein digests, for example), usually at concentrations much higher than are normally available *in situ*. Two key assumptions made with these common medium formulations are that (i) microbes will use only the relevant constituents and any remaining compounds will have minor or no effect on microbial growth and (ii) microbes grow optimally in the laboratory when nutrient availability is much greater than their half-saturation constant (47). While many commonly studied microbes have the capacity to grow on complex, high-nutrient formulations, environmental nucleic acid data inform us that the vast majority of Earth’s microbes remain uncultured (46, 48). Our results indicate that the concentration of nutrients in a growth medium may be as important as the constituents of the growth medium for cultivation of uncultured environmental microbes.

Numerous studies have demonstrated that dilute growth medium is superior to substrate-rich growth medium for the isolation of novel soil microbes (33, 34, 49, 50). However, the physiological explanation of why low-nutrient medium facilitates the growth of diverse microbes, or high nutrient concentrations inhibit the growth of some taxa, remains unclear. One possible explanation for these concentration-dependent effects may be that growth medium formulations applied at high concentrations contain large amounts of inhibitory substances—substances that are reduced to noninhibitory levels in dilute medium formulations. For example, a key amino acid transporter in Chlamydia trachomatis can be blocked by nonessential amino acids, preventing the transport of required amino acids, resulting in growth inhibition (51). A similar phenomenon was demonstrated in the extreme marine oligotroph “*Candidatus* Pelagibacter ubique,” where alanine was conditionally required for cell division but abolished growth at higher concentrations (52). Furthermore, reactive oxygen species can be produced during the autoclaving of nutrient-rich medium that either directly inhibit growth or combine with organics in the medium to form inhibitory compounds (53, 54). Finally, growth inhibition may be the result of misbalanced regulation of growth or accumulation of nutrient storage structures (poly-β-hydroxybutyrate, for example), ultimately leading to cell lysis (55). A better understanding of the mechanism(s) that enables growth on low-nutrient medium—or prevents growth on high-nutrient medium—may help us design better strategies for isolating uncultivated lineages. Critically, the collection of cultures we describe here, which were isolated on medium with identical constituents applied at different concentrations, is a first step toward an experimental method capable of addressing these questions.

While several of the taxa we isolated were abundant microbial members of the shallow subsurface microbial community (Fig. 4), other isolates were rare or not identified in the cultivation-independent soil microbial community. The cultivation of additional microbial phylotypes that were not observed in molecular analyses of the same samples has been observed (56, 57). The dilution-to-extinction approach we used here favors the cultivation of abundant microbes in a given sample (40), such that the isolation of rarer taxa or taxa that were not observed in the original sample was unexpected. There are several possible explanations for this observation. First, the buoyant density separation protocol that we used to separate cells may have introduced biases. Nycodenz cell separation approaches do not recover all microbial cells from soil, and the separable fraction can be compositionally distinct from the nonseparated soils (58). These effects could potentially skew the proportions of microbes that were diluted into microtiter plate wells. Alternatively, the absence of a particular taxon in a soil microbiome analysis may be the result of insufficient sequencing depth (56). Moreover, the “universal” primers used in the soil microbiome analysis (59) may not have primed DNA from some of the divergent lineages we cultured as efficiently as other phylotypes in the soil microbiome, resulting in either underrepresentation of these phylotypes in the original community or no amplification at all. We do not have sufficient evidence indicating which of these scenarios may explain our ability to culture cells that were not apparent in the microbiome analysis. Finally, as is true in any microbial cultivation experiment, there were many taxa that we did not isolate. In particular, these soils contained high relative abundances of *Verrucomicrobia* related to “*Candidatus* Udaeobacter copiosus” (60) and *Acidobacteria* (subgroup 6), which belong to highly sought-after lineages of uncultured microbes (61). The reasons for not isolating these (and other) lineages are numerous but may be the result of inappropriate medium composition (62, 63), toxic compounds in the cell separation constituents, long doubling times (>∼6 days), or dormancy (reviewed in reference 64).

We provide evidence that microbes cultured from oligotrophic soils on low-nutrient medium may have reduced nucleic acid content relative to microbes isolated on richer medium (Fig. 5). Depending on the taxa in question, and their effective population size, microbial genome reduction can be driven by either genetic drift or “streamlining” selection (reviewed in reference 65). Genome streamlining is strongly linked with microbial oligotrophy in free-living aquatic microbes as a mechanism to reduce the overhead cost of replication in periodically nutrient-limited environments (reviewed in reference 21). However, direct evidence for genome streamlining in terrestrial microbes has been elusive. For example, metagenome-assembled genomes of abundant and ubiquitous uncultured *Verrucomicrobia* suggest that some lineages may contain reduced genome sizes (60). A more recent study showed that fire-affected warm soils selected for groups of microbes with significantly smaller genomes than cooler soils (66). Yet, there are few definitive ways to identify the growth preferences of taxa with reduced genomes short of culturing them and studying their growth dynamics under controlled conditions. The appearance of reduced nucleic acid content in cultures isolated on ASM-low may be an indication that genome reduction may be a successful life strategy for soil oligotrophs. Alternative explanations for the apparent differences in nucleic acid content in microbes cultured in ASM-low may be that (i) the ploidy of stationary-phase cells grown in ASM-low may be lower than those isolated in ASM-high, (ii) unknown cellular constituents of cells grown in ASM-low may quench SYBR green I fluorescence in the assay conditions we used, or (iii) cells isolated in ASM-high may form small microaggregates that are not completely dispersed prior to flow cytometry.

The development of cultivation techniques emphasizing the high-throughput and sensitive detection of microbial growth on low-nutrient medium revolutionized the field of aquatic microbial ecology by culturing microbes that were previously “unculturable” using standard techniques (38, 39, 67–69). Here, we show that similar cultivation principles facilitate the cultivation of abundant soil microbes. We demonstrate that, in addition to scrutinizing the nutritional composition of a given growth medium, the concentration of growth substrates in the growth medium must also be considered. Although we do not yet understand the mechanism of substrate-induced growth inhibition, there is evidence that this phenomenon is widespread and may impede laboratory cultivation efforts. Future studies to deduce the molecular mechanisms of substrate-induced growth inhibition will likely lead to new cultivation approaches that will allow us to isolate abundant free-living oligotrophic microbes.

## MATERIALS AND METHODS

### Soil source and nutrient analysis.

Fresh soil samples were collected from a soil pit on 16 August 2017 at the Oracle Ridge site in the Catalina Jemez Critical Zone Observatory (coordinates: 32.45 N, −110.74 W, elevation 2,103 m). The mean annual precipitation at Oracle Ridge is 87 cm year^−1^, and the mean annual surface temperature is 12°C (70). The soils were Typic Ustorthent (70). The dominant vegetation at the site is ponderosa pine (Pinus ponderosa), with sparse Douglas fir (Pseudotsuga menziesii). We collected ∼300-g subsamples from 0 cm, 10 cm, 20 cm, 30 cm, 40 cm, and 55 cm depths. Soils were kept cool with ice packs for <4 h while in transit to the laboratory. At the laboratory, the soils were sieved to 2 mm and kept at 4°C for 50 days, at which point cells were separated from mineral soil. Standard soil chemical analyses were performed at the Colorado State University Soil Water and Plant Testing Laboratory using their standard protocols. We analyzed the microbial community composition at each depth (see “Soil microbial community analysis” below) and conducted cultivation experiments from the 55 cm soil sample.

### Soil microbial community analysis.

We extracted DNA from 1.0 g subsamples (*n* = 3) using MoBio PowerSoil DNA extraction kits according to the manufacturer’s instructions. We amplified 16S rRNA gene fragments using 515F-Y (5′-TATGGTAATTGTGTGYCAGCMGCCGCGGTAA-3′) and 926R (5′-AGTCAGTCAGGGCCGYCAATTCMTTTRAGT-3′) (71). PCR products were purified using the QIAquick PCR purification kit (Qiagen, Germantown, MD) per manufacturer’s specifications. Cleaned products were quantified using Tecan fluorometric methods (Tecan Group, Mannedorf, Switzerland), normalized, and pooled for Illumina MiSeq sequencing using custom sequencing primers and the MiSeq Reagent v2 500 cycle kit (Illumina, San Diego, CA) according to the manufacturer’s protocols. We identified phylotypes based on the generation of *de novo* operational taxonomic units (OTUs) from raw Illumina sequence reads using the UPARSE pipeline at a stringency of 97% identity (72). Paired-end reads were trimmed of adapter sequences, barcodes, and primers prior to assembly. We discarded low-quality and singleton sequences and dereplicated the remaining sequences before calculating relative abundances. Chimera filtering of the sequences was completed during clustering, while taxonomy was assigned to the OTUs with mothur (73) using version 123 of the SILVA 16S rRNA database (74) as the reference. We generated OTU and taxonomy assignment tables for subsequent analyses.

### Cell separation.

Cells were separated from sieved soils using buoyant density centrifugation with Nycodenz modified from reference 37 to isolate viable cells. Briefly, we added 0.5 g wet soil to 44.8 ml of cell extraction buffer (137.5 mM NaCl, 26.78 mM tetrasodium pyrophosphate, and 0.27% [vol/vol] Tween 80). The soil-buffer slurry was vortexed for 30 s and shaken horizontally on a platform shaker for 2 h at 4°C. We layered 15-ml aliquots of this soil-buffer slurry over 10.0 ml of 80% (wt/vol) Nycodenz solution in 50 mM tetrasodium pyrophosphate. We used 50 ml Nalgene Oak Ridge high-speed polycarbonate centrifuge tubes for buoyant density centrifugations. Tubes containing the soil-buffer solution with Nycodenz were centrifuged at 17,000 × *g* for 30 min at 16°C. We extracted three 0.5-ml aliquots from the resulting buoyant density preparation at a location of ∼25 mm above the bottom of the tube (coincident with the approximate level of the top of the Nycodenz solution) to sterile microcentrifuge tubes containing 1.0 ml 137.5 mM NaCl. The microcentrifuge tubes containing Nycodenz/NaCl were vortexed and centrifuged for 20 min at 17,000 × *g*. The resulting cell pellets were resuspended in 137.5 mM NaCl, pooled, and stored at 4°C.

### Medium design rationale.

The ASM media were custom designed to facilitate the growth of a broad range of soil chemoheterotrophic microbes (see Table S1 in the supplemental material). Both ASM-high and ASM-low were buffered with phosphate. To this, we added minerals at concentrations derived from an “artificial rainwater” recipe (75), trace elements as described in trace element solution SL-10 with the addition of LaCl_3_, and vitamins as described elsewhere (52). We added heterotrophic growth substrates that included 21 amino acids and a diverse range of simple carbon substrates including 2-C substrates (glycerol and acetate), 3-C substrates (pyruvate), 4-C substrates (succinate, butyrate, and isobutyrate), 5-C substrates (ribose and valerate), a 6-C substrate (glucose), an 8-C substrate (*N*-acetylglucosamine), and a 10-C substrate (decanoic acid) (Table S1). We also added several polymeric growth substrates including pectin, methylcellulose, alginate, starch, and xylan (Table S1). We calculated the added carbon amount to be ∼200 mg C liter^−1^ for ASM-high and ∼2 mg C liter^−1^ for ASM-low.

### Dilution-to-extinction.

An aliquot of cells extracted from the buoyant density separation was fixed with 1.75% (final [vol/vol]) formaldehyde and stained with SYBR green I (final stain concentration was a 1:4,000 dilution of commercial stock) for 3.5 h at room temperature in the dark. Cells were enumerated using a Millipore Guava flow cytometer, as described elsewhere (52). We diluted cells into artificial subterranean medium (ASM)-high or ASM-low nutrient medium (Table S1) to a density of 5 cells ml^−1^ and aliquoted 1.0 ml of the dilute cell suspension into the wells of 2 ml polytetrafluoroethylene 96-well microtiter plates (Cowie Technology, New Castle, DE) so that on average each well contained 5 cells. Plates were covered with plastic lids that allowed air circulation and incubated at 16°C in the dark under aerobic conditions. We screened the dilution-to-extinction plates for growth by fixing (1.75% formaldehyde) and staining (1:4,000 dilution of commercial SYBR green I stock) aliquots for 18 h in the dark at room temperature and counting by flow cytometry (EMD-Millipore Guava EasyCyte), as described previously (52). We screened plates for growth at 4 and 11 weeks after inoculation. Positive cultures were defined as cultures that exceeded 1.0 × 10^4^ cells ml^−1^.

### Actual and theoretical culturability estimates.

Culturability estimates were determined by the equation *V* = −ln(1 − *p*)/*X*, where *V* is the estimated culturability, *p* is the proportion of inoculated cultivation chambers that displayed measurable growth (number of chambers positive for growth/total number of chambers inoculated), and *X* is the number of cells added to each cultivation chamber as estimated from dilutions (40). The number of pure cultures (*û*) was estimated as follows: *û =* −*n*(1 − *p*) × ln(1 − *p*), where *n* is the number of inoculated growth chambers and *p* is the proportion of inoculated wells displaying growth (40).

### Culture transfer and storage.

We subcultured positive growth chambers into 25 ml of the respective growth medium (ASM-high or ASM-low) in acid-washed, sterile polycarbonate flasks and incubated them at 16°C. At the time of transfer, we assigned cultures a unique internal identification number for our Arizona Culture Collection (AZCC). Flasks were monitored for growth every other week for 2 months. Flasks displaying growth within 2 months were cryopreserved in 10% glycerol and stored at −80°C. If no growth appeared within 2 months, the cultures were discarded and the assigned AZCC number was retired.

### Mean fluorescence calculations.

We calculated the mean fluorescence of each culture from the subcultures grown in 25 ml volumes at 12 to 15 weeks after inoculating. Culture aliquots were fixed and stained for 15 to 18 h as described above under “Dilution-to-extinction.” We manually gated histograms of the intensity of SYBR green I fluorescence (in arbitrary units) and extracted the mean fluorescence of the gated peak for each culture using the GuavaSoft software package. “Best hit” genomes were determined by subjecting the full-length 16S rRNA gene sequence of our isolates to a BLAST search against the NCBI Microbial Genomes database using web-blast (76). We extracted the total genome length from each best-hit genome.

### Culture identification.

Cultures were identified by full-length 16S rRNA gene sequencing. Briefly, we filtered 5 to 10 ml of cell biomass from 25 ml cultures onto 0.2 μm pore size Supor filters and extracted DNA using a Qiagen PowerSoil DNA extraction kit according to the manufacturer’s instructions. We amplified full-length 16S rRNA genes from the resulting DNA using the 27F-1492R primer set (27F, 5′-AGAGTTTGATCMTGGCTCAG-3′; 1492R, 5′-ACCTTGTTACGACTT-3′ [77]). The reaction mix consisted of Promega’s GoTaq HotStart 2× PCR master mix with final concentrations of 0.4 μM 27F and 0.4 μM 1492R primers, and 1 to 11.5 μl of template DNA, in a total reaction volume of 25 μl. The thermocycling profile was once at 94°C for 10 min followed by 36 cycles of 94°C for 45 s, 50°C for 90 s, and 72°C for 90 s, and a single 72°C extension for 10 min. The resulting amplicons were cleaned and Sanger sequenced from both the 27F and 1492R primers by Eurofins Genomics (Louisville, KY, USA) using their standard techniques. Sequences were curated using 4Peaks (78) and Geneious Prime v2019.0.1 (79). Reads were trimmed and assembled using the moderate setting in Geneious. Forward and reverse Sanger PCR reads that failed to build a full-length 16S rRNA gene with these metrics were considered “mixed” cultures and not analyzed further.

### Culture taxonomy and determination of taxonomic differences across growth medium formulations.

High-quality full-length 16S rRNA gene sequences from the cultures were used to assign taxonomy and reconstruct phylogenetic relationships. We assigned taxonomy to all assembled 16S rRNA gene sequences using the SILVA database SINA aligner v128 (80). A Shapiro-Wilk test of normality was conducted in base R (81) on the distribution of SILVA genus assignments from both medium types. After concluding the data were nonparametric, we performed a Kruskal-Wallis test in R (assigned genus ∼ medium type). We performed a *post hoc* analysis (Dunn test, in R) to determine whether culturability within a phylum varied by growth medium type.

### Taxonomic selection for phylogenetic reconstruction.

To reconstruct a phylogeny of full-length 16S rRNA genes, our culture sequences were compared to NCBI’s Microbial Genomes and environmental sequence databases using web-blast (76). The top five hits for each sequence from each NCBI database were chosen based on the highest percent coverage and lowest E value score and included in the reconstruction. Escherichia coli K-12 was used as the outgroup of the alphaproteobacterial phylogeny, and Bacillus subtilis was used as the outgroup for the actinobacterial tree. These sequences aligned with MAFFT (82) with turn checking enabled. The alignment was then trimmed using trimAl (83) with the “automated1” setting to optimize sequence trimming for maximum-likelihood (ML) phylogenetic analyses. We reconstructed phylogenetic relationships from this trimmed alignment in the CIPRES Gateway (84). Maximum-likelihood (ML) trees were constructed using IQ-TREE with 10,000 ultrafast bootstrap trees and Bayesian Information Criterion to select the best-fit nucleic acid substitution model (85, 86). For *Actinobacteria*, we used the SYM+R10 model, and for *Alphaproteobacteria*, we used the GTR+F+I+G4 model. After an initial round of ML trees, sequence alignments were heuristically curated with IQ-TREE to eliminate sequences that appeared in the tree more than once. Finalized ML trees were then imported into the ARB environment (87), where any duplicate sequences from our AZCC cultures were added to the ML trees through ARB’s quick add parsimony function. Final trees were visualized with FigTree (88).

### Environmental contextualization of AZCC isolates.

We matched the AZCC isolate full-length 16S rRNA gene sequences against a database of the clustered OTUs from the shallow soil depth profile samples (see “Soil microbial community analysis” above) using the usearch_global command (89) at a stringency of ≥97% identity, in both strand orientations, with maxaccepts = 1 and maxrejects = 0.

### Data availability.

Full-length Sanger-sequenced 16S rRNA gene sequences are available on NCBI GenBank under accession numbers MK875836 to MK875967. Illumina data from the 55-cm Oracle Ridge community are available on the NCBI SRA under accession numbers SRR9172130 to SRR9172198.

## References

[1] Chaparro JM, Sheflin AM, Manter DK, Vivanco JM 2012 Manipulating the soil microbiome to increase soil health and plant fertility. Biol Fertil Soils 48:489–499. doi:10.1007/s00374-012-0691-4.

[2] Long PE, Williams KH, Hubbard SS, Banfield JF 2016 Microbial metagenomics reveals climate-relevant subsurface biogeochemical processes. Trends Microbiol 24:600–610. doi:10.1016/j.tim.2016.04.006. 27156744

[3] Fernández-Luqueño F, Valenzuela-Encinas C, Marsch R, Martínez-Suárez C, Vázquez-Núñez E, Dendooven L 2011 Microbial communities to mitigate contamination of PAHs in soil—possibilities and challenges: a review. Environ Sci Pollut Res Int 18:12–30. doi:10.1007/s11356-010-0371-6. 20623198

[4] Tiedje JM, Asuming-Brempong S, Nüsslein K, Marsh TL, Flynn SJ 1999 Opening the black box of soil microbial diversity. Appl Soil Ecol 13:109–122. doi:10.1016/S0929-1393(99)00026-8.

[5] Jobbagy EG, Jackson RB 2000 The vertical distribution of soil organic carbon and its relation to climate and vegetation. Ecol Appl 10:423–436. doi:10.1890/1051-0761(2000)010[0423:TVDOSO]2.0.CO;2.

[6] Fierer N, Schimel JP, Holden PA 2003 Variations in microbial community composition through two soil depth profiles. Soil Biol Biochem 35:167–176. doi:10.1016/S0038-0717(02)00251-1.

[7] Ajwa HA, Rice CW, Sotomayor D 1998 Carbon and nitrogen mineralization in tallgrass prairie and agricultural soil profiles. Soil Sci Soc Am J 62:942–951. doi:10.2136/sssaj1998.03615995006200040014x.

[8] Jobbágy EG, Jackson RB 2001 The distribution of soil nutrients with depth: global patterns and the imprint of plants. Biogeochemistry 53:51–77. doi:10.1023/A:1010760720215.

[9] Balesdent J, Basile-Doelsch I, Chadoeuf J, Cornu S, Derrien D, Fekiacova Z, Hatté C 2018 Atmosphere-soil carbon transfer as a function of soil depth. Nature 559:599–602. doi:10.1038/s41586-018-0328-3. 29995858

[10] Hillel D 1980 Fundamentals of soil physics. Academic Press, New York, NY.

[11] Brewer TE, Aronson EL, Arogyaswamy K, Billings SA, Botthoff JK, Campbell AN, Dove NC, Fairbanks D, Gallery RE, Hart SC, Kaye J, King G, Logan G, Lohse KA, Maltz MR, Mayorga E, O’Neill C, Owens SM, Packman A, Pett-Ridge J, Plante AF, Richter DD, Silver WL, Yang WH, Fierer N 2019 Ecological and genomic attributes of novel bacterial taxa that thrive in subsurface soil horizons. mBio 10:e01318-19. doi:10.1128/mBio.01318-19. 31575762 PMC6775450

[12] Eilers KG, Debenport S, Anderson S, Fierer N 2012 Digging deeper to find unique microbial communities: the strong effect of depth on the structure of bacterial and archaeal communities in soil. Soil Biol Biochem 50:58–65. doi:10.1016/j.soilbio.2012.03.011.

[13] Blume E, Bischoff M, Reichert JM, Moorman T, Konopka A, Turco RF 2002 Surface and subsurface microbial biomass, community structure and metabolic activity as a function of soil depth and season. Appl Soil Ecol 20:171–181. doi:10.1016/S0929-1393(02)00025-2.

[14] Spohn M, Klaus K, Wanek W, Richter A 2016 Microbial carbon use efficiency and biomass turnover times depending on soil depth—implications for carbon cycling. Soil Biol Biochem 96:74–81. doi:10.1016/j.soilbio.2016.01.016.

[15] Overmann J, Abt B, Sikorski J 2017 Present and future of culturing bacteria. Annu Rev Microbiol 71:711–730. doi:10.1146/annurev-micro-090816-093449. 28731846

[16] Gao Y, Wu M 2018 Free-living bacterial communities are mostly dominated by oligotrophs. bioRxiv 350348 https://www.biorxiv.org/content/10.1101/350348v1.

[17] Morita RY 1997 Bacteria in oligotrophic environments: starvation-survival lifestyle, 1st ed. Chapman and Hall, New York, NY.

[18] Schut F, Prins RA, Gottschal JC 1997 Oligotrophy and pelagic marine bacteria: facts and fiction. Aquat Microb Ecol 12:177–202. doi:10.3354/ame012177.

[19] Kuznetsov SI, Dubinina GA, Lapteva NA 1979 Biology of oligotrophic bacteria. Annu Rev Microbiol 33:377–387. doi:10.1146/annurev.mi.33.100179.002113. 386927

[20] Cho J-C, Giovannoni SJ 2004 Cultivation and growth characteristics of a diverse group of oligotrophic marine gammaproteobacteria. Appl Environ Microbiol 70:432–440. doi:10.1128/aem.70.1.432-440.2004. 14711672 PMC321273

[21] Giovannoni SJ, Cameron Thrash J, Temperton B 2014 Implications of streamlining theory for microbial ecology. ISME J 8:1553–1565. doi:10.1038/ismej.2014.60. 24739623 PMC4817614

[22] Lauro FM, McDougald D, Thomas T, Williams TJ, Egan S, Rice S, DeMaere MZ, Ting L, Ertan H, Johnson J, Ferriera S, Lapidus A, Anderson I, Kyrpides N, Munk AC, Detter C, Han CS, Brown MV, Robb FT, Kjelleberg S, Cavicchioli R 2009 The genomic basis of trophic strategy in marine bacteria. Proc Natl Acad Sci U S A 106:15527–15533. doi:10.1073/pnas.0903507106. 19805210 PMC2739866

[23] Poindexter JS 1981 Oligotrophy: fast and famine existence. Adv Microb Ecol 5:63–90. doi:10.1007/978-1-4615-8306-6_2.

[24] Giovannoni SJ 2017 SAR11 bacteria: the most abundant plankton in the oceans. Annu Rev Mar Sci 9:231–255. doi:10.1146/annurev-marine-010814-015934. 27687974

[25] Swan BK, Tupper B, Sczyrba A, Lauro FM, Martinez-Garcia M, Gonzalez JM, Luo H, Wright JJ, Landry ZC, Hanson NW, Thompson BP, Poulton NJ, Schwientek P, Acinas SG, Giovannoni SJ, Moran MA, Hallam SJ, Cavicchioli R, Woyke T, Stepanauskas R 2013 Prevalent genome streamlining and latitudinal divergence of planktonic bacteria in the surface ocean. Proc Natl Acad Sci U S A 110:11463–11468. doi:10.1073/pnas.1304246110. 23801761 PMC3710821

[26] Santoro AE, Dupont CL, Richter RA, Craig MT, Carini P, McIlvin MR, Yang Y, Orsi WD, Moran DM, Saito MA 2015 Genomic and proteomic characterization of “*Candidatus* Nitrosopelagicus brevis”: an ammonia-oxidizing archaeon from the open ocean. Proc Natl Acad Sci U S A 112:1173–1178. doi:10.1073/pnas.1416223112. 25587132 PMC4313803

[27] Noell SE, Giovannoni SJ 2019 SAR11 bacteria have a high affinity and multifunctional glycine betaine transporter. Environ Microbiol 21:2559–2575. doi:10.1111/1462-2920.14649. 31090982

[28] Carini P, Dupont CL, Santoro AE 2018 Patterns of thaumarchaeal gene expression in culture and diverse marine environments. Environ Microbiol 20:2112–2124. doi:10.1111/1462-2920.14107. 29626379

[29] Könneke M, Bernhard AE, de la Torre JR, Walker CB, Waterbury JB, Stahl DA 2005 Isolation of an autotrophic ammonia-oxidizing marine archaeon. Nature 437:543–546. doi:10.1038/nature03911. 16177789

[30] Santoro AE, Casciotti KL 2011 Enrichment and characterization of ammonia-oxidizing archaea from the open ocean: phylogeny, physiology and stable isotope fractionation. ISME J 5:1796–1808. doi:10.1038/ismej.2011.58. 21562601 PMC3197170

[31] Hu SJ, Van Bruggen AHC, Grünwald NJ 1999 Dynamics of bacterial populations in relation to carbon availability in a residue-amended soil. Appl Soil Ecol 13:21–30. doi:10.1016/S0929-1393(99)00015-3.

[32] Saito A, Mitsui H, Hattori R, Minamisawa K, Hattori T 1998 Slow-growing and oligotrophic soil bacteria phylogenetically close to Bradyrhizobium japonicum. FEMS Microbiol Ecol 25:277–286. doi:10.1111/j.1574-6941.1998.tb00480.x.

[33] Davis KER, Joseph SJ, Janssen PH 2005 Effects of growth medium, inoculum size, and incubation time on culturability and isolation of soil bacteria. Appl Environ Microbiol 71:826–834. doi:10.1128/AEM.71.2.826-834.2005. 15691937 PMC546801

[34] Joseph SJ, Hugenholtz P, Sangwan P, Osborne CA, Janssen PH 2003 Laboratory cultivation of widespread and previously uncultured soil bacteria. Appl Environ Microbiol 69:7210–7215. doi:10.1128/aem.69.12.7210-7215.2003. 14660368 PMC309996

[35] Zengler K 2017 Protocols for high-throughput isolation and cultivation, p 27–35. *In* McGenity TJ, Timmis KN, Balbina N (ed), Hydrocarbon and lipid microbiology protocols: isolation and cultivation. Springer, Berlin, Germany.

[36] Jiang CY, Dong L, Zhao JK, Hu X, Shen C, Qiao Y, Zhang X, Wang Y, Ismagilov RF, Liu SJ, Du W 2016 High-throughput single-cell cultivation on microfluidic streak plates. Appl Environ Microbiol 82:2210–2218. doi:10.1128/AEM.03588-15. 26850294 PMC4807504

[37] Khalili B, Weihe C, Kimball S, Schmidt KT, Martiny J 2019 Optimization of a method to quantify soil bacterial abundance by flow cytometry. mSphere 4:e00435-19. doi:10.1128/mSphere.00435-19. 31597718 PMC6796974

[38] Stingl U, Tripp HJ, Giovannoni SJ 2007 Improvements of high-throughput culturing yielded novel SAR11 strains and other abundant marine bacteria from the Oregon coast and the Bermuda Atlantic Time Series study site. ISME J 1:361–371. doi:10.1038/ismej.2007.49. 18043647

[39] Connon SA, Giovannoni SJ 2002 High-throughput methods for culturing microorganisms in very-low-nutrient media. Appl Environ Microbiol 68:3878–3885. doi:10.1128/aem.68.8.3878-3885.2002. 12147485 PMC124033

[40] Button DK, Schut F, Quang P, Martin R, Robertson BR 1993 Viability and isolation of marine bacteria by dilution culture: theory, procedures, and initial results. Appl Environ Microbiol 59:881–891. doi:10.1128/AEM.59.3.881-891.1993. 16348896 PMC202203

[41] Tahon G, Willems A 2017 Isolation and characterization of aerobic anoxygenic phototrophs from exposed soils from the Sør Rondane Mountains, East Antarctica. Syst Appl Microbiol 40:357–369. doi:10.1016/j.syapm.2017.05.007. 28705596

[42] Wang Y, Hammes F, Boon N, Chami M, Egli T 2009 Isolation and characterization of low nucleic acid (LNA)-content bacteria. ISME J 3:889–902. doi:10.1038/ismej.2009.46. 19421234

[43] Proctor CR, Besmer MD, Langenegger T, Beck K, Walser JC, Ackermann M, Bürgmann H, Hammes F 2018 Phylogenetic clustering of small low nucleic acid-content bacteria across diverse freshwater ecosystems. ISME J 12:1344–1359. doi:10.1038/s41396-018-0070-8. 29416124 PMC5932017

[44] Liu J, Hao Z, Ma L, Ji Y, Bartlam M, Wang Y 2016 Spatio-temporal variations of high and low nucleic acid content bacteria in an exorheic river. PLoS One 11:e0153678. doi:10.1371/journal.pone.0153678. 27082986 PMC4833404

[45] Liu J, Li JQ, Feng L, Cao H, Cui Z 2010 An improved method for extracting bacteria from soil for high molecular weight DNA recovery and BAC library construction. J Microbiol 48:728–733. doi:10.1007/s12275-010-0139-1. 21221926

[46] Steen AD, Crits-Christoph A, Carini P, DeAngelis KM, Fierer N, Lloyd KG, Cameron Thrash J 2019 High proportions of bacteria and archaea across most biomes remain uncultured. ISME J 13:3126–3130. doi:10.1038/s41396-019-0484-y. 31388130 PMC6863901

[47] Monod J 1949 The growth of bacterial cultures. Annu Rev Microbiol 3:371–394. doi:10.1146/annurev.mi.03.100149.002103.

[48] Lloyd KG, Steen AD, Ladau J, Yin J, Crosby L 2018 Phylogenetically novel uncultured microbial cells dominate earth microbiomes. mSystems 3:e00055-18. doi:10.1128/mSystems.00055-18. 30273414 PMC6156271

[49] Sait M, Hugenholtz P, Janssen PH 2002 Cultivation of globally distributed soil bacteria from phylogenetic lineages previously only detected in cultivation-independent surveys. Environ Microbiol 4:654–666. doi:10.1046/j.1462-2920.2002.00352.x. 12460273

[50] Janssen PH, Yates PS, Grinton BE, Taylor PM, Sait M 2002 Improved culturability of soil bacteria and isolation in pure culture of novel members of the divisions Acidobacteria, Actinobacteria, Proteobacteria, and Verrucomicrobia. Appl Environ Microbiol 68:2391–2396. doi:10.1128/aem.68.5.2391-2396.2002. 11976113 PMC127570

[51] Braun PR, Al-Younes H, Gussmann J, Klein J, Schneider E, Meyer TF 2008 Competitive inhibition of amino acid uptake suppresses chlamydial growth: involvement of the chlamydial amino acid transporter BrnQ. J Bacteriol 190:1822–1830. doi:10.1128/JB.01240-07. 18024516 PMC2258683

[52] Carini P, Steindler L, Beszteri S, Giovannoni SJ 2013 Nutrient requirements for growth of the extreme oligotroph “Candidatus Pelagibacter ubique” HTCC1062 on a defined medium. ISME J 7:592–602. doi:10.1038/ismej.2012.122. 23096402 PMC3578571

[53] Tanaka T, Kawasaki K, Daimon S, Kitagawa W, Yamamoto K, Tamaki H, Tanaka M, Nakatsu CH, Kamagata Y 2014 A hidden pitfall in the preparation of agar media undermines microorganism cultivability. Appl Environ Microbiol 80:7659–7666. doi:10.1128/AEM.02741-14. 25281372 PMC4249246

[54] Carlsson J, Nyberg G, Wrethén J 1978 Hydrogen peroxide and superoxide radical formation in anaerobic broth media exposed to atmospheric oxygen. Appl Environ Microbiol 36:223–229. doi:10.1128/AEM.36.2.223-229.1978. 29560 PMC291205

[55] Baxter M, Sieburth JM 1984 Metabolic and ultrastructural response to glucose of two eurytrophic bacteria isolated from seawater at different enriching concentrations. Appl Environ Microbiol 47:31–38. doi:10.1128/AEM.47.1.31-38.1984. 6696421 PMC239607

[56] Lau JT, Whelan FJ, Herath I, Lee CH, Collins SM, Bercik P, Surette MG 2016 Capturing the diversity of the human gut microbiota through culture-enriched molecular profiling. Genome Med 8:72. doi:10.1186/s13073-016-0327-7. 27363992 PMC4929786

[57] Diakite A, Dubourg G, Dione N, Afouda P, Bellali S, Ngom II, Valles C, Million M, Levasseur A, Cadoret F, Lagier JC, Raoult D 2019 Extensive culturomics of 8 healthy samples enhances metagenomics efficiency. PLoS One 14:e0223543. doi:10.1371/journal.pone.0223543. 31634343 PMC6802823

[58] Portillo MC, Leff JW, Lauber CL, Fierer N 2013 Cell size distributions of soil bacterial and archaeal taxa. Appl Environ Microbiol 79:7610–7617. doi:10.1128/AEM.02710-13. 24077710 PMC3837822

[59] Walters W, Hyde ER, Berg-Lyons D, Ackermann G, Humphrey G, Parada A, Gilbert JA, Jansson JK, Caporaso JG, Fuhrman JA, Apprill A, Knight R 2015 Improved bacterial 16S rRNA gene (V4 and V4-5) and fungal internal transcribed spacer marker gene primers. mSystems 1:e00009-15. doi:10.1128/mSystems.00009-15. 27822518 PMC5069754

[60] Brewer TE, Handley KM, Carini P, Gilbert JA, Fierer N 2016 Genome reduction in an abundant and ubiquitous soil bacterium “Candidatus Udaeobacter copiosus.” Nat Microbiol 2:16198. doi:10.1038/nmicrobiol.2016.198. 27798560

[61] Carini P 2019 A “cultural” renaissance: genomics breathes new life into an old craft. mSystems 4:e00092-19. doi:10.1128/mSystems.00092-19. 31219785 PMC6533372

[62] Olsen RA, Bakken LR 1987 Viability of soil bacteria: optimization of plate-counting technique and comparison between total counts and plate counts within different size groups. Microb Ecol 13:59–74. doi:10.1007/BF02014963. 24213103

[63] Aagot N, Nybroe O, Nielsen P, Johnsen K 2001 An altered pseudomonas diversity is recovered from soil by using nutrient-poor pseudomonas-selective soil extract media. Appl Environ Microbiol 67:5233–5239. doi:10.1128/AEM.67.11.5233-5239.2001. 11679350 PMC93295

[64] Lennon JT, Jones SE 2011 Microbial seed banks: the ecological and evolutionary implications of dormancy. Nat Rev Microbiol 9:119–130. doi:10.1038/nrmicro2504. 21233850

[65] Batut B, Knibbe C, Marais G, Daubin V 2014 Reductive genome evolution at both ends of the bacterial population size spectrum. Nat Rev Microbiol 12:841–850. doi:10.1038/nrmicro3331. 25220308

[66] Sorensen JW, Dunivin TK, Tobin TC, Shade A 2019 Ecological selection for small microbial genomes along a temperate-to-thermal soil gradient. Nat Microbiol 4:55–61. doi:10.1038/s41564-018-0276-6. 30397342

[67] Rappe MS, Connon SA, Vergin KL, Giovannoni SJ 2002 Cultivation of the ubiquitous SAR11 marine bacterioplankton clade. Nature 418:630–633. doi:10.1038/nature00917. 12167859

[68] Kim S, Kang I, Seo JH, Cho JC 2019 Culturing the ubiquitous freshwater actinobacterial acI lineage by supplying a biochemical ‘helper’ catalase. ISME J 13:2252–2263. doi:10.1038/s41396-019-0432-x. 31073214 PMC6775976

[69] Salcher MM, Schaefle D, Kaspar M, Neuenschwander SM, Ghai R 2019 Evolution in action: habitat-transition leads to genome-streamlining in Methylophilaceae. ISME J 13:2764–2777. doi:10.1038/s41396-019-0471-3. 31292537 PMC6794327

[70] Lybrand RA, Rasmussen C 2015 Quantifying climate and landscape position controls on soil development in semiarid ecosystems. Soil Sci Soc Am J 79:104–116. doi:10.2136/sssaj2014.06.0242.

[71] Parada AE, Needham DM, Fuhrman JA 2016 Every base matters: assessing small subunit rRNA primers for marine microbiomes with mock communities, time series and global field samples. Environ Microbiol 18:1403–1414. doi:10.1111/1462-2920.13023. 26271760

[72] Edgar RC 2013 UPARSE: highly accurate OTU sequences from microbial amplicon reads. Nat Methods 10:996–998. doi:10.1038/nmeth.2604. 23955772

[73] Schloss PD, Westcott SL, Ryabin T, Hall JR, Hartmann M, Hollister EB, Lesniewski RA, Oakley BB, Parks DH, Robinson CJ, Sahl JW, Stres B, Thallinger GG, Van Horn DJ, Weber CF 2009 Introducing mothur: open-source, platform-independent, community-supported software for describing and comparing microbial communities. Appl Environ Microbiol 75:7537–7541. doi:10.1128/AEM.01541-09. 19801464 PMC2786419

[74] Quast C, Pruesse E, Yilmaz P, Gerken J, Schweer T, Yarza P, Peplies J, Glöckner FO 2013 The SILVA ribosomal RNA gene database project: improved data processing and web-based tools. Nucleic Acids Res 41:D590–D596. doi:10.1093/nar/gks1219. 23193283 PMC3531112

[75] Kox MAR, Lüke C, Fritz C, van den Elzen E, van Alen T, Op den Camp HJM, Lamers LPM, Jetten MSM, Ettwig KF 2016 Effects of nitrogen fertilization on diazotrophic activity of microorganisms associated with Sphagnum magellanicum. Plant Soil 406:83–100. doi:10.1007/s11104-016-2851-z.

[76] Johnson M, Zaretskaya I, Raytselis Y, Merezhuk Y, McGinnis S, Madden TL 2008 NCBI BLAST: a better web interface. Nucleic Acids Res 36:W5–W9. doi:10.1093/nar/gkn201. 18440982 PMC2447716

[77] Lane DJ 1991 16S/23S rRNA sequencing, p 115–175. In Stackebrandt E, Goodfellow M (ed), Nucleic acid techniques in bacterial systematics. John Wiley and Sons, Chichester, United Kingdom.

[78] Griekspoor A, Groothuis T 2004 4Peaks, 1.8. Nucleobytes, Aalsmeer, Netherlands.

[79] Biomatters, Ltd. 2019 Geneious prime, v2019.0.1. Biomatters, Ltd, Auckland, New Zealand.

[80] Pruesse E, Peplies J, Glöckner FO 2012 SINA: accurate high-throughput multiple sequence alignment of ribosomal RNA genes. Bioinformatics 28:1823–1829. doi:10.1093/bioinformatics/bts252. 22556368 PMC3389763

[81] R Core Team. 2014 R: a language and environment for statistical computing, 3.1.1. R Foundation for Statistical Computing, Vienna, Austria.

[82] Katoh K, Standley DM 2013 MAFFT multiple sequence alignment software version 7: improvements in performance and usability. Mol Biol Evol 30:772–780. doi:10.1093/molbev/mst010. 23329690 PMC3603318

[83] Capella-Gutiérrez S, Silla-Martínez JM, Gabaldón T 2009 trimAl: a tool for automated alignment trimming in large-scale phylogenetic analyses. Bioinformatics 25:1972–1973. doi:10.1093/bioinformatics/btp348. 19505945 PMC2712344

[84] Miller MA, Pfeiffer W, Schwartz T 2010 Creating the CIPRES Science Gateway for inference of large phylogenetic trees *In* 2010 Gateway Computing Environments Workshop. IEEE, New York, NY.

[85] Minh BQ, Nguyen MAT, Von Haeseler A 2013 Ultrafast approximation for phylogenetic bootstrap. Mol Biol Evol 30:1188–1195. doi:10.1093/molbev/mst024. 23418397 PMC3670741

[86] Nguyen L-T, Schmidt HA, von Haeseler A, Minh BQ 2015 IQ-TREE: a fast and effective stochastic algorithm for estimating maximum-likelihood phylogenies. Mol Biol Evol 32:268–274. doi:10.1093/molbev/msu300. 25371430 PMC4271533

[87] Ludwig W, Strunk O, Westram R, Richter L, Meier H, Yadhukumar, Buchner A, Lai T, Steppi S, Jobb G, Förster W, Brettske I, Gerber S, Ginhart AW, Gross O, Grumann S, Hermann S, Jost R, König A, Liss T, Lüßmann R, May M, Nonhoff B, Reichel B, Strehlow R, Stamatakis A, Stuckmann N, Vilbig A, Lenke M, Ludwig T, Bode A, Schleifer K 2004 ARB: a software environment for sequence data. Nucleic Acids Res 32:1363–1371. doi:10.1093/nar/gkh293. 14985472 PMC390282

[88] Rambaut A, Drummond A 2016. FigTree 1.4.3.

[89] Edgar RC 2010 Search and clustering orders of magnitude faster than BLAST. Bioinformatics 26:2460–2461. doi:10.1093/bioinformatics/btq461. 20709691

[90] Carini P, Marsden PJ, Leff JW, Morgan EE, Strickland MS, Fierer N 2016 Relic DNA is abundant in soil and obscures estimates of soil microbial diversity. Nat Microbiol 2:16242. doi:10.1038/nmicrobiol.2016.242. 27991881

[91] Delgado-Baquerizo M, Oliverio AM, Brewer TE, Benavent-González A, Eldridge DJ, Bardgett RD, Maestre FT, Singh BK, Fierer N 2018 A global atlas of the dominant bacteria found in soil. Science 359:320–325. doi:10.1126/science.aap9516. 29348236

[92] Eichorst SA, Kuske CR, Schmidt TM 2011 Influence of plant polymers on the distribution and cultivation of bacteria in the phylum Acidobacteria. Appl Environ Microbiol 77:586–596. doi:10.1128/AEM.01080-10. 21097594 PMC3020536

