# Supplementary material for: Influence of Substrate Concentration on the Culturability of Heterotrophic Soil Microbes Isolated by High-Throughput Dilution-to-Extinction Cultivation
Source: mSphere. 2020 Jan 29;5(1):e00024-20. doi: 10.1128/mSphere.00024-20 (PMC6992367; doi:10.1128/mSphere.00024-20)
Supplement: FIG S2 [file mSphere.00024-20-sf002.pdf]

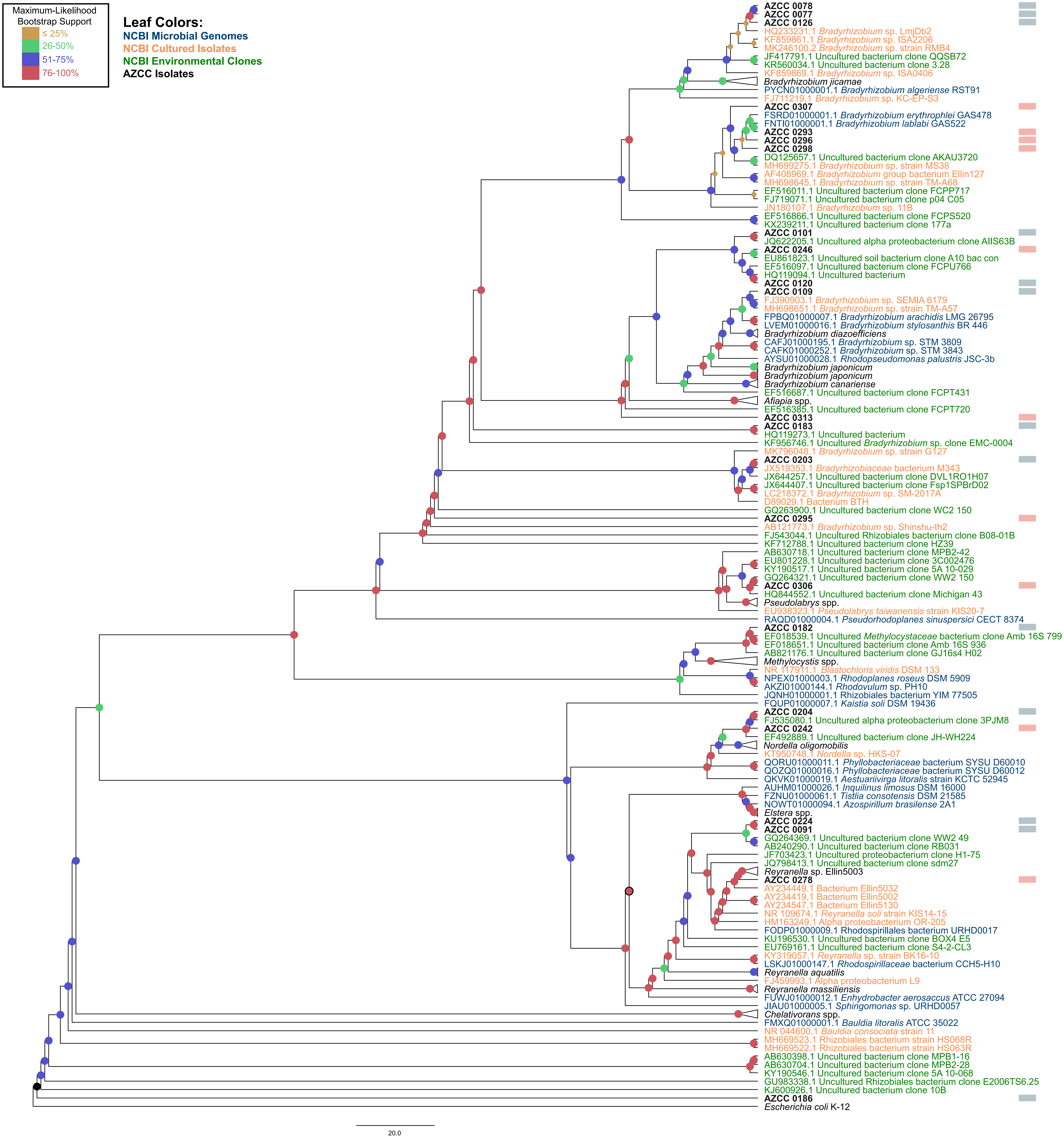

Maximum-Likelihood Bootstrap Support
≤ 25%
26-50%
51-75%
76-100%
Leaf Colors:
NCBI Microbial Genomes
NCBI Cultured Isolates
NCBI Environmental Clones
AZCC Isolates
AZCC 0078
AZCC 0077
AZCC 0126
HQ233231.1 Bradyrhizobium sp. LmjDb2
KF859861.1 Bradyrhizobium sp. ISA2206
MK246100.2 Bradyrhizobium sp. strain RMB4
JF417791.1 Uncultured bacterium clone QQSB72
KR560034.1 Uncultured bacterium clone 3.28
KF859869.1 Bradyrhizobium sp. ISA0406
Bradyrhizobium jicamae
PYCN01000001.1 Bradyrhizobium algeriense RST91
FJ711219.1 Bradyrhizobium sp. KC-EP-S3
AZCC 0307
FSRD01000001.1 Bradyrhizobium erythrophlei GAS478
FNTI01000001.1 Bradyrhizobium lablabi GAS522
AZCC 0293
AZCC 0296
AZCC 0298
DQ125657.1 Uncultured bacterium clone AKAU3720
MH699275.1 Bradyrhizobium sp. strain MS38
AF408969.1 Bradyrhizobium group bacterium Ellin127
MH698645.1 Bradyrhizobium sp. strain TM-A68
EF516011.1 Uncultured bacterium clone FCPP717
FJ719071.1 Uncultured bacterium clone p04 C05
JN180107.1 Bradyrhizobium sp. 11B
EF516866.1 Uncultured bacterium clone FCPS520
KX239211.1 Uncultured bacterium clone 177a
AZCC 0101
JQ622205.1 Uncultured alpha proteobacterium clone AIIS63B
AZCC 0246
EU861823.1 Uncultured soil bacterium clone A10 bac con
EF516097.1 Uncultured bacterium clone FCPU766
HQ119094.1 Uncultured bacterium
AZCC 0120
AZCC 0109
FJ390903.1 Bradyrhizobium sp. SEMIA 6179
MH698651.1 Bradyrhizobium sp. strain TM-A57
FPBQ01000007.1 Bradyrhizobium arachidis LMG 26795
LVEM01000016.1 Bradyrhizobium stylosanthis BR 446
Bradyrhizobium diazoefficiens
CAFJ01000195.1 Bradyrhizobium sp. STM 3809
CAFK01000252.1 Bradyrhizobium sp. STM 3843
AYSU01000028.1 Rhodopseudomonas palustris JSC-3b
Bradyrhizobium japonicum
Bradyrhizobium japonicum
Bradyrhizobium canariense
EF516687.1 Uncultured bacterium clone FCPT431
Afiapia spp.
EF516385.1 Uncultured bacterium clone FCPT720
AZCC 0313
AZCC 0183
HQ119273.1 Uncultured bacterium
KF956746.1 Uncultured Bradyrhizobium sp. clone EMC-0004
MK796048.1 Bradyrhizobium sp. strain G127
AZCC 0203
JX519353.1 Bradyrhizobiaceae bacterium M343
JX644257.1 Uncultured bacterium clone DVL1RO1H07
JX644407.1 Uncultured bacterium clone Fsp1SPBrD02
LC218372.1 Bradyrhizobium sp. SM-2017A
D89029.1 Bacterium BTH
GQ263900.1 Uncultured bacterium clone WC2 150
AZCC 0295
AB121773.1 Bradyrhizobium sp. Shinshu-th2
FJ543044.1 Uncultured Rhizobiales bacterium clone B08-01B
KF712788.1 Uncultured bacterium clone HZ39
AB630718.1 Uncultured bacterium clone MPB2-42
EU801228.1 Uncultured bacterium clone 3C002476
KY190517.1 Uncultured bacterium clone 5A 10-029
GQ264321.1 Uncultured bacterium clone WW2 150
AZCC 0306
HQ844552.1 Uncultured bacterium clone Michigan 43
Pseudolabrys spp.
EU938323.1 Pseudolabrys taiwanensis strain KIS20-7
RAQD01000004.1 Pseudorhodoplanes sinuspersici CECT 8374
AZCC 0182
EF018539.1 Uncultured Methylocystaceae bacterium clone Amb 16S 799
EF018651.1 Uncultured bacterium clone Amb 16S 936
AB821176.1 Uncultured bacterium clone GJ16s4 H02
Methylocystis spp.
NR 117911.1 Blastochloris viridis DSM 133
NPEX01000003.1 Rhodoplanes roseus DSM 5909
AKZI01000144.1 Rhodovulum sp. PH10
JQNH01000001.1 Rhizobiales bacterium YIM 77505
FQUP01000007.1 Kaistia soli DSM 19436
AZCC 0204
FJ535080.1 Uncultured alpha proteobacterium clone 3PJM8
AZCC 0242
EF492889.1 Uncultured bacterium clone JH-WH224
Nordella oligomobilis
KT950748.1 Nordella sp. HKS-07
QORU01000011.1 Phyllobacteriaceae bacterium SYSU D60010
QOZQ01000016.1 Phyllobacteriaceae bacterium SYSU D60012
QKVK01000019.1 Aestuariivirga litoralis strain KCTC 52945
AUHM01000026.1 Inquilinus limosus DSM 16000
FZNU01000061.1 Tistlia consotensis DSM 21585
NOWT01000094.1 Azospirillum brasilense 2A1
Elstera spp.
AZCC 0224
AZCC 0091
GQ264369.1 Uncultured bacterium clone WW2 49
AB240290.1 Uncultured bacterium clone RB031
JF703423.1 Uncultured proteobacterium clone H1-75
JQ798413.1 Uncultured bacterium clone sdm27
Reyranella sp. Ellin5003
AZCC 0278
AY234449.1 Bacterium Ellin5032
AY234419.1 Bacterium Ellin5002
AY234547.1 Bacterium Ellin5130
NR 109674.1 Reyranella soli strain KIS14-15
HM163249.1 Alpha proteobacterium OR-205
FODP01000009.1 Rhodospirillales bacterium URHD0017
KU196530.1 Uncultured bacterium clone BOX4 E5
EU769161.1 Uncultured bacterium clone S4-2-CL3
KY319057.1 Reyranella sp. strain BK16-10
LSKJ01000147.1 Rhodospirillaceae bacterium CCH5-H10
Reyranella aquatilis
FJ459993.1 Alpha proteobacterium L9
Reyranella massiliensis
FUWJ01000012.1 Enhydrobacter aerosaccus ATCC 27094
JIAU01000005.1 Sphingomonas sp. URHD0057
Chelativorans spp.
FMXQ01000001.1 Bauldia litoralis ATCC 35022
NR 044600.1 Bauldia consociata strain 11
MH669523.1 Rhizobiales bacterium strain HS068R
MH669522.1 Rhizobiales bacterium strain HS063R
AB630398.1 Uncultured bacterium clone MPB1-16
AB630704.1 Uncultured bacterium clone MPB2-28
KY190546.1 Uncultured bacterium clone 5A 10-068
GU983338.1 Uncultured Rhizobiales bacterium clone E2006TS6.25
KJ600926.1 Uncultured bacterium clone 10B
AZCC 0186
Escherichia coli K-12
20.0
