# Supplementary material for: Influence of Substrate Concentration on the Culturability of Heterotrophic Soil Microbes Isolated by High-Throughput Dilution-to-Extinction Cultivation
Source: mSphere. 2020 Jan 29;5(1):e00024-20. doi: 10.1128/mSphere.00024-20 (PMC6992367; doi:10.1128/mSphere.00024-20)
Supplement: FIG S3 [file mSphere.00024-20-sf003.pdf]

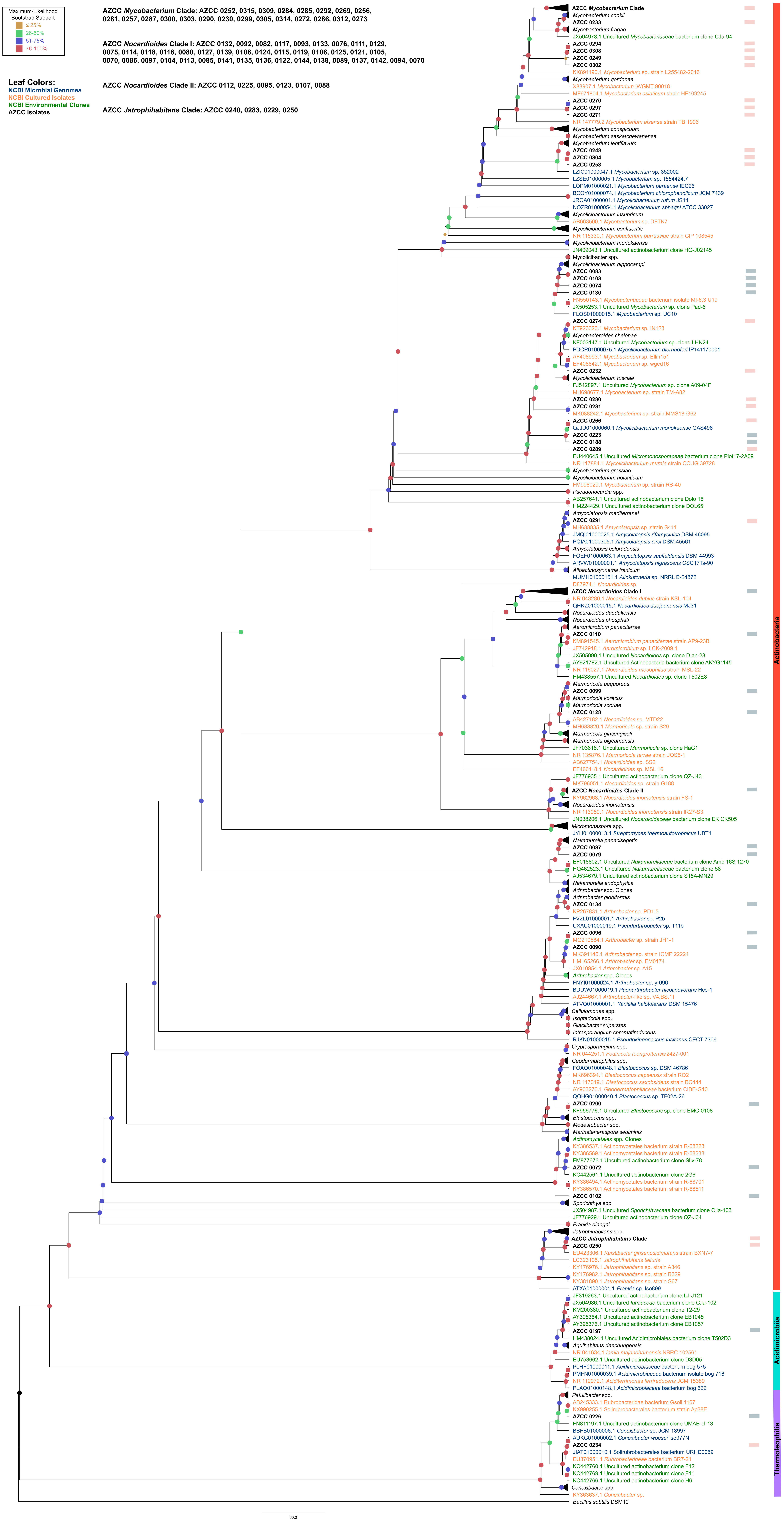

Maximum-Likelihood Bootstrap Support
≤ 25%
26-50%
51-75%
76-100%
Leaf Colors:
NCBI Microbial Genomes
NCBI Cultured Isolates
NCBI Environmental Clones
AZCC Isolates
AZCC Mycobacterium Clade: AZCC 0252, 0315, 0309, 0284, 0285, 0292, 0269, 0256, 0281, 0257, 0287, 0300, 0303, 0290, 0230, 0299, 0305, 0314, 0272, 0286, 0312, 0273
AZCC Nocardioides Clade I: AZCC 0132, 0092, 0082, 0117, 0093, 0133, 0076, 0111, 0129, 0075, 0114, 0118, 0116, 0080, 0127, 0139, 0108, 0124, 0115, 0119, 0106, 0125, 0121, 0105, 0070, 0086, 0097, 0104, 0113, 0085, 0141, 0135, 0136, 0122, 0144, 0138, 0089, 0137, 0142, 0094, 0070
AZCC Nocardioides Clade II: AZCC 0112, 0225, 0095, 0123, 0107, 0088
AZCC Jatrophihabitans Clade: AZCC 0240, 0283, 0229, 0250
AZCC Mycobacterium Clade
Mycobacterium cookii
AZCC 0233
Mycobacterium fragae
JX504978.1 Uncultured Mycobacteriaceae bacterium clone C.la-94
AZCC 0294
AZCC 0308
AZCC 0249
AZCC 0302
KX891190.1 Mycobacterium sp. strain L255482-2016
Mycobacterium gordonae
X88907.1 Mycobacterium IWGMT 90018
MF671804.1 Mycobacterium asiaticum strain HF109245
AZCC 0270
AZCC 0297
AZCC 0271
NR 147779.2 Mycobacterium alsense strain TB 1906
Mycobacterium conspicuum
Mycobacterium saskatchewanense
Mycobacterium lentiflavum
AZCC 0248
AZCC 0304
AZCC 0253
LZIC01000047.1 Mycobacterium sp. 852002
LZSE01000005.1 Mycobacterium sp. 1554424.7
LQPM01000021.1 Mycobacterium paraense IEC26
BCQY01000074.1 Mycobacterium chlorophenolicum JCM 7439
JROA01000001.1 Mycolicibacterium rufum JS14
NOZR01000054.1 Mycolicibacterium sphagni ATCC 33027
Mycolicibacterium insubricum
AB663500.1 Mycobacterium sp. DFTK7
Mycolicibacterium confluentis
NR 115330.1 Mycobacterium barrassiae strain CIP 108545
Mycolicibacterium moriokaense
JN409043.1 Uncultured actinobacterium clone HG-J02145
Mycolicibacter spp.
Mycolicibacterium hippocampi
AZCC 0083
AZCC 0103
AZCC 0074
AZCC 0130
FN550143.1 Mycobacteriaceae bacterium isolate MI-6.3 U19
JX505253.1 Uncultured Mycobacterium sp. clone Pad-6
FLQS01000015.1 Mycobacterium sp. UC10
AZCC 0274
KT923323.1 Mycobacterium sp. IN123
Mycobacteroides chelonae
KF003147.1 Uncultured Mycobacterium sp. clone LHN24
PDCR01000075.1 Mycolicibacterium diernhoferi IP141170001
AF408993.1 Mycobacterium sp. Ellin151
EF408842.1 Mycobacterium sp. wged16
AZCC 0232
Mycolicibacterium tusciae
FJ542897.1 Uncultured Mycobacterium sp. clone A09-04F
MH698677.1 Mycobacterium sp. strain TM-A82
AZCC 0280
AZCC 0231
MK088242.1 Mycobacterium sp. strain MMS18-G62
AZCC 0266
QJJU01000060.1 Mycolicibacterium moriokaense GAS496
AZCC 0223
AZCC 0188
AZCC 0289
EU440645.1 Uncultured Micromonosporaceae bacterium clone Plot17-2A09
NR 117884.1 Mycolicibacterium murale strain CCUG 39728
Mycobacterium grossiae
Mycolicibacterium holsaticum
FM998029.1 Mycobacterium sp. strain RS-40
Pseudonocardia spp.
AB257641.1 Uncultured actinobacterium clone Dolo 16
HM224429.1 Uncultured actinobacterium clone DOL65
Amycolatopsis mediterranei
AZCC 0291
MH688835.1 Amycolatopsis sp. strain S411
JMQI01000025.1 Amycolatopsis rifamycinica DSM 46095
PQIA01000305.1 Amycolatopsis circi DSM 45561
Amycolatopsis coloradensis
FOEF01000063.1 Amycolatopsis saalfeldensis DSM 44993
ARVW01000001.1 Amycolatopsis nigrescens CSC17Ta-90
Alloactinosynnema iranicum
MUMH01000151.1 Allokutzneria sp. NRRL B-24872
D87974.1 Nocardioides sp.
AZCC Nocardioides Clade I
NR 043280.1 Nocardioides dubius strain KSL-104
QHKZ01000015.1 Nocardioides daejeonensis MJ31
Nocardioides daedukensis
Nocardioides phosphati
Aeromicrobium panaciterrae
AZCC 0110
KM891545.1 Aeromicrobium panaciterrae strain AP9-23B
JF742918.1 Aeromicrobium sp. LCK-2009.1
JX505090.1 Uncultured Nocardioides sp. clone D.an-23
AY921782.1 Uncultured Actinobacteria bacterium clone AKYG1145
NR 116027.1 Nocardioides mesophilus strain MSL-22
HM438557.1 Uncultured Nocardioides sp. clone T502E8
Marmoricola aequoreus
AZCC 0099
Marmoricola korecus
Marmoricola scoriae
AZCC 0128
AB427182.1 Nocardioides sp. MTD22
MH688820.1 Marmoricola sp. strain S29
Marmoricola ginsengisoli
Marmoricola bigeumensis
JF703618.1 Uncultured Marmoricola sp. clone HaG1
NR 135876.1 Marmoricola terrae strain JOS5-1
AB627754.1 Nocardioides sp. SS2
EF466118.1 Nocardioides sp. MSL 16
JF776935.1 Uncultured actinobacterium clone QZ-J43
MK796051.1 Nocardioides sp. strain G188
AZCC Nocardioides Clade II
KY962968.1 Nocardioides iriomotensis strain FS-1
Nocardioides iriomotensis
NR 113050.1 Nocardioides iriomotensis strain IR27-S3
JN038206.1 Uncultured Nocardioidaceae bacterium clone EK CK505
Micromonospora spp.
JYIJ01000013.1 Streptomyces thermoautotrophicus UBT1
Nakamurella panacisegetis
AZCC 0087
AZCC 0079
EF018802.1 Uncultured Nakamurellaceae bacterium clone Amb 16S 1270
HQ462523.1 Uncultured Nakamurellaceae bacterium clone 58
AJ534679.1 Uncultured actinobacterium clone S15A-MN29
Nakamurella endophytica
Arthrobacter spp. Clones
Arthrobacter globiformis
AZCC 0134
KP267831.1 Arthrobacter sp. PD1.5
FVZL01000001.1 Arthrobacter sp. P2b
UXAU01000019.1 Pseudarthrobacter sp. T11b
AZCC 0096
MG210584.1 Arthrobacter sp. strain JH1-1
AZCC 0090
MK391146.1 Arthrobacter sp. strain ICMP 22224
HM165266.1 Arthrobacter sp. EM0174
JX010954.1 Arthrobacter sp. A15
Arthrobacter spp. Clones
FNYI01000024.1 Arthrobacter sp. yr096
BDDW01000019.1 Paenarthrobacter nicotinovorans Hce-1
AJ244667.1 Arthrobacter-like sp. V4.BS.11
ATVQ01000001.1 Yaniella halotolerans DSM 15476
Cellulomonas spp.
Isoptericola spp.
Glaciibacter superstes
Intrasporangium chromatireducens
RJKN01000015.1 Pseudokineococcus lusitanus CECT 7306
Cryptosporangium spp.
NR 044251.1 Fodinicola feengrottensis 2427-001
Geodermatophilus spp.
FOAO01000048.1 Blastococcus sp. DSM 46786
MK696394.1 Blastococcus capsensis strain RQ2
NR 117019.1 Blastococcus saxobsidens strain BC444
AY903276.1 Geodermatophilaceae bacterium CIBE-G10
QOHG01000040.1 Blastococcus sp. TF02A-26
AZCC 0200
KF956776.1 Uncultured Blastococcus sp. clone EMC-0108
Blastococcus spp.
Modestobacter spp.
Marinatenerasporа sediminis
Actinomycetales spp. Clones
KY386537.1 Actinomycetales bacterium strain R-68223
KY386569.1 Actinomycetales bacterium strain R-68238
FM877676.1 Uncultured actinobacterium clone Sliv-78
AZCC 0072
KC442561.1 Uncultured actinobacterium clone 2G6
KY386494.1 Actinomycetales bacterium strain R-68701
KY386570.1 Actinomycetales bacterium strain R-68511
AZCC 0102
Sporichthya spp.
JX504987.1 Uncultured Sporichthyaceae bacterium clone C.la-103
JF776929.1 Uncultured actinobacterium clone QZ-J34
Frankia elaegni
Jatrophihabitans spp.
AZCC Jatrophihabitans Clade
AZCC 0250
EU423306.1 Kaistibacter ginsenosidimutans strain BXN7-7
LC323105.1 Jatrophihabitans telluris
KY176976.1 Jatrophihabitans sp. strain A346
KY176982.1 Jatrophihabitans sp. strain B329
KY381890.1 Jatrophihabitans sp. strain S67
ATXA01000001.1 Frankia sp. Iso899
JF319263.1 Uncultured actinobacterium clone LJ-J121
JX504986.1 Uncultured Iamiaceae bacterium clone C.la-102
KM200380.1 Uncultured actinobacterium clone T2-29
AY395364.1 Uncultured actinobacterium clone EB1045
AY395376.1 Uncultured actinobacterium clone EB1057
AZCC 0197
HM438024.1 Uncultured Acidimicrobiales bacterium clone T502D3
Aquihabitans daechungensis
NR 041634.1 Iamia majanohamensis NBRC 102561
EU753662.1 Uncultured actinobacterium clone D3D05
PLHF01000011.1 Acidimicrobiaceae bacterium bog 575
PMFN01000039.1 Acidimicrobiaceae bacterium isolate bog 716
NR 112972.1 Aciditerrimonas ferrireducens JCM 15389
PLAQ01000148.1 Acidimicrobiaceae bacterium bog 622
Patulibacter spp.
AB245333.1 Rubrobacteridae bacterium Gsoil 1167
KX990255.1 Solirubrobacterales bacterium strain Ap38E
AZCC 0226
FN811197.1 Uncultured actinobacterium clone UMAB-cl-13
BBFB01000006.1 Conexibacter sp. JCM 18997
AUKG01000002.1 Conexibacter woesei Iso977N
AZCC 0234
JIAT01000010.1 Solirubrobacterales bacterium URHD0059
EU370951.1 Rubrobacterineae bacterium BR7-21
KC442760.1 Uncultured actinobacterium clone F12
KC442769.1 Uncultured actinobacterium clone F11
KC442766.1 Uncultured actinobacterium clone H6
Conexibacter spp.
KY363637.1 Conexibacter sp.
Bacillus subtilis DSM10
60.0
Actinobacteria
Acidimicrobiia
Thermoleophilia
